# Systemic CSF1R Targeting Depletes Pathogenic MPS Bubs and Ameliorates Psoriasis via PPARα-mediated Resolution

**DOI:** 10.7150/thno.128248

**Published:** 2026-01-30

**Authors:** Zhen-Jia Lin, Ying Li, Yangyinhui Yu, Mei-Jia Fang, Ying Xiong, Rui Xu, Zhuo Wang, Jun Zhang, Ya-Nan Xu, Jun-Ya Wan, Xiang Ji, Yu-Fan Zheng, Kai-Lang Zhang, Ming Wei, Jun-Tao Zou, Li-Xuan Jia, Hui Zhang, Chang-Lin Li, Li-Jun Zhou, Zhi Tan

**Affiliations:** 1 Department of Human Anatomy and Physiology and Pain Research Center, Zhongshan School of Medicine and Guangdong Province Key Laboratory of Brain Function and Disease, Sun Yat-sen University, No.74, 2nd Zhongshan Road, Yuexiu District, Guangzhou 510080, China.; 2 Department of Anesthesiology, The Third Affiliated Hospital of Sun Yat-sen University, No. 600, Tianhe Road, Tianhe District, Guangzhou 510630, China.; 3 Department of Dermatology, The First Affiliated Hospital of Sun Yat-sen University, No.58, 2nd Zhongshan Road, Yuexiu District, Guangzhou 510080, China.; 4 Department of Pathology, The First Affiliated Hospital of Sun Yat-sen University, No.58, 2nd Zhongshan Road, Yuexiu District, Guangzhou 510080, China.; 5 Department of Anesthesiology and Pain Clinic, First Affiliated Hospital of Sun Yat-sen University, No.58, 2nd Zhongshan Road, Yuexiu District, Guangzhou 510080, China.; 6 Department of Anesthesiology, The Affiliated Guangdong Second Provincial General Hospital of Jinan University, No.466, Xingang Middle Road, Haizhu District, Guangzhou 510317, China.; 7 Guangdong Institute of Intelligence Science and Technology, Hengqin 519031, China.

**Keywords:** psoriasis, mononuclear phagocyte system (MPS), CSF1R, PPARα, inflammation

## Abstract

**Rationale:** Psoriasis features persistent activation of the mononuclear phagocyte system (MPS), yet the subset-specific pathogenic roles of colony-stimulating factor 1 receptor (CSF1R) remain undefined. We aimed to identify pathogenic CSF1R^high^ MPS subsets, characterize their ligand-receptor circuits, and define the CSF1R-PPARα axis in disease pathogenesis.

**Methods:** By integrating human single-cell and spatial transcriptomics with murine imiquimod (IMQ)-induced psoriasis models, we employed genetic and pharmacologic interventions to achieve our aims.

**Results:** We found a pathologic CSF1R^high^ MPS population was selectively expanded, forming localized cytokine hubs enriched for TNF-α, IL-1β, and IL-23. Ligand mapping showed CSF1 upregulation amplified MPS activation via autocrine loops. Systemic CSF1R targeting dismantled skin-blood MPS circuits and depleted pathogenic hubs, suppressing pro-inflammatory cytokines more effectively than local blockade. Mechanistically, CSF1R activation directly suppressed PPARα. Critically, the anti-inflammatory effect of CSF1R inhibition was abrogated by PPARα antagonism, demonstrating a non-redundant, downstream role for PPARα. Consequently, CSF1R suppression releases PPARα-mediated resolution programs. Pathogenic CSF1R^high^ MPS hubs sustain inflammation through ligand-driven expansion and PPARα suppression.

**Conclusions:** Our work delineates a unidirectional CSF1R-PPARα pathogenic axis and demonstrates that systemic CSF1R targeting is required to disrupt this circuit, providing a mechanistic foundation for a novel treatment strategy.

## Introduction

Psoriasis is a heterogeneous chronic immune-mediated disorder affecting over 60 million individuals worldwide 1. Biologics targeting the IL-23/IL-17 axis have revolutionized disease management and now constitute the cornerstone of induction and maintenance therapy for plaque psoriasis (PV) 2. However, important unmet needs persist, particularly in severe or unstable subtypes such as generalized pustular psoriasis (GPP) and erythrodermic psoriasis (EP), which exhibit neutrophil-dominant inflammation, systemic cytokine release, and profound disruption of cutaneous immune homeostasis 3. While IL-17/23-directed therapies achieve high clinical response rates during continuous treatment, accumulating evidence indicates that they primarily suppress downstream inflammatory effector pathways rather than eliminate upstream disease-initiating cellular programs 4, 5. In particular, pathogenic populations within the mononuclear phagocyte system (MPS) can persist in lesional skin and reinitiate inflammation upon treatment withdrawal 6. Thus, the limitation of current biologics lies not in insufficient on-treatment efficacy, but in incomplete immune reprogramming at the level of non-lymphoid inflammatory reservoirs. Achieving durable disease control may therefore require therapeutic strategies that extend beyond cytokine neutralization to directly target upstream MPS-centered pathogenic circuits.

The MPS—comprising monocytes, macrophages, and dendritic cells—plays a central role in orchestrating psoriatic inflammation through spatially organized pathogenic hubs 7. Epidermal MPS cells such as Langerhans cells (LCs) license IL-17^⁺^ T cell expansion, whereas dermal MPS clusters secrete TNF-α and IL-23 to recruit neutrophils 2, 8, 9. However, the lack of markers distinguishing pathogenic from homeostatic MPS subsets has hindered the development of precision-targeted interventions.

Colony-stimulating factor 1 receptor (CSF1R), a tyrosine kinase receptor essential for MPS survival and differentiation 10, is expressed under homeostasis via its ligands CSF1 and IL-34 11-13. CSF1R blockade alleviates inflammation in IL-23-driven murine psoriasis models 14, yet whether CSF1R preferentially marks pathogenic MPS subsets in human disease and how its signaling amplifies inflammation remain unresolved. Beyond regulating cell survival, CSF1R signaling has been implicated in macrophage metabolic and inflammatory programming 15, suggesting the existence of downstream regulatory checkpoints.

Peroxisome proliferator-activated receptor α (PPARα) is a key nuclear receptor that promotes lipid metabolism and inflammation resolution in macrophages 16, 17. Although PPARα expression is reduced in psoriatic skin 18, the upstream mechanisms driving this suppression are unknown.

Here, we integrate spatial multi-omics with compartment-specific interventions to: 1) map pathogenic CSF1R^high^ MPS hub dynamics in human psoriasis and murine models; 2) compare the efficacy of systemic versus local CSF1R targeting; and 3) definitively establish PPARα as a critical mechanistic endpoint of CSF1R-driven pathology using functional loss-of-function experiments. We identify CSF1R^high^ MPS subsets as master regulators of treatment-resistant inflammation and demonstrate that systemic CSF1R targeting effectively depletes these hubs by releasing endogenous PPARα-mediated resolution programs. These findings establish the CSF1R-PPARα axis as a core pathogenic circuit and demonstrate that its disruption via systemic targeting establishes a foundational strategy for achieving durable disease control.

## Materials and Methods

### Sex as a biological variable

Human samples were selected to include both male and female individuals with an approximately balanced sex distribution. Sex-stratified analyses confirmed that the core findings and conclusions were consistent between sexes. In the IMQ-induced psoriasis mouse model, only female mice were used, as male mice frequently exhibit aggressive behavior that can interfere with skin lesion development and longitudinal assessment, a limitation commonly recognized in this model.

### Clinical studies

This study utilized a retrospective cohort of 48 individuals recruited from the Dermatology Outpatient Clinic of the First Affiliated Hospital, Sun Yat-sen University. The cohort comprised 32 patients with active psoriasis, including plaque psoriasis (PV, n = 16), generalized pustular psoriasis (GPP, n = 8), and erythrodermic psoriasis (EP, n = 8), as well as age- and sex-matched healthy controls (HC, n = 16). All patients had discontinued systemic therapy for at least 4 weeks and topical treatments for at least 72 h prior to sample collection.

Peripheral blood samples (HC: n = 12, PV/GPP/EP: n = 8/group) were collected in EDTA tubes and analyzed within 2 h using a Sysmex XN-550 hematology analyzer. Absolute counts of monocytes, granulocytes, neutrophils, lymphocytes, and eosinophils were recorded, and monocyte-to-lymphocyte ratio (MLR) and neutrophil-to-lymphocyte ratio (NLR) were calculated.

Skin punch biopsies (4 mm) were obtained from lesional skin of patients and normal-appearing skin of healthy controls under local anesthesia. For immunohistochemistry (IHC) and immunofluorescence (IF) analyses, tissues were fixed in 10% neutral-buffered formalin for 24 h, paraffin-embedded, and sectioned at 3 μm (HC: IHC n = 12, IF n = 4, PV/GPP/EP: IHC n = 8/group, PV IF n = 6). Additional snap-frozen specimens (lesional skin from 16 PV patients and normal skin from 8 HC individuals) were stored at -80 °C for Western blot (WB) analysis.

Detailed demographic and clinical characteristics of all human skin and blood samples are summarized in Table S1-2.

### Animals

Female C57BL/6J WT mice (8-10-week-old, weighing 18-22 g) were obtained from Laboratory Animal Center, Sun Yat-sen University (Guangzhou, China). Two transgenic strains were used: 1)* Csf1r*^+/-^ mice (Stock#: C57BL/6-Trim47^tm1cyagen^, Cyagen Biosciences Inc., China); 2) *Cx3cr1*^CreER/+^*; Csf1r*^fl/fl^ mice, kindly provided by Prof. Jiyun Peng (Institute of Brain Science, Hangzhou Normal University). All mice were housed under specific pathogen-free conditions with a 12 h light/dark cycle, maintained at 23 ± 2 °C and 50-60% humidity.

### CSF1R⁺ MPS depletion

Tamoxifen-induced recombination using the *Cx3cr1*^CreER^ has been extensively validated to efficiently target circulating monocytes, monocyte-derived macrophages, and tissue-resident CX3CR1^⁺^ macrophage and dendritic cell populations, while largely sparing granulocytes and lymphoid lineages 19-21. Accordingly, *Cx3cr1^CreER^; Csf1r^fl/fl^* mice were employed to genetically ablate CSF1R across the peripheral CX3CR1^⁺^ myeloid continuum relevant to psoriatic inflammation.

Importantly, although *Cx3cr1*^CreER^ does not uniformly recombine all dendritic cell subsets, it preferentially targets CSF1R^⁺^ macrophages, inflammatory monocytes, and monocyte-derived DCs, which constitute the dominant effector and organizing populations within psoriatic lesions. This targeting profile closely matches the disease-relevant CSF1R^high^ myeloid populations identified by our single-cell and spatial transcriptomic analyses, thereby maximizing functional relevance while minimizing off-target recombination in non-MPS compartments 19, 22.

For systemic MPS depletion (G-CSF1R CKO), *Cx3cr1^CreER^; Csf1r^fl/fl^* mice received intraperitoneal injections of tamoxifen (20 mg/mL in corn oil, 150 mg/kg) once daily for 5 consecutive days. Psoriasis modeling began 72 h after the final injection to ensure efficient recombination.

For local MPS depletion (L-CSF1R CKO), mice received subcutaneous injections of endoxifen (2 mg/mL in saline, 5 mg/kg) 24 h before IMQ induction and daily thereafter. Control groups received equivalent volumes of corn oil or saline via the same routes and schedules.

### IMQ-induced psoriasis mouse models

Psoriasiform dermatitis was induced by daily topical application of 5% imiquimod cream (62.5 mg on a shaved 2 × 2 cm² dorsal area) for 5 consecutive days 23. For studies of subclinical inflammation, a low-dose IMQ regimen ((L-IMQ, 20.8 mg/day) was applied 24, with Vaseline as vehicle control. In the psoriasis recurrence model, mice underwent three cycles of IMQ treatment (5 days/cycle) separated by 6-week remission periods (Figure 2J-K). Disease severity was assessed daily by: 1) Change in skin fold thickness (Δ, mm) measured with digital calipers; 2) Psoriasis Area and Severity Index (PASI) scoring, including erythema, scaling, and thickness (each graded 0-4; total scores 0-12).

For mechanistic analyses, tissue and cell samples were collected at predefined time points according to the biological processes interrogated. Early innate immune infiltration was assessed at day 1.5 by flow cytometry. Transcriptomic analyses and recurrence (“recall”) responses were evaluated at day 2.5, enabling direct comparison between primary and repeated IMQ challenges. Day 4.5 was used as the peak inflammatory time point for histological, immunostaining, and endpoint analyses.

### RNA sequencing and bioinformatics

Murine skin bulk RNA-seq was performed on 2.5d skin lesions from 4 groups: WT CON (n = 3), WT IMQ (n = 4), *Csf1r*^+/-^ CON (n = 4), and *Csf1r*^+/-^ IMQ (n = 4). The back skin of the experimental mice was taken after anesthesia. Total RNA in tissues was isolated using TRIzol reagent (Invitrogen) and then sent to Biomarker (Beijing Biomarker Technologies Co., LTD.) for bulk RNA-seq analysis. The sequencing libraries were generated using the NEBNext Ultra Directional RNA Library Prep Kit (New England Biolabs) and sequenced on an Illumina NovaSeq 6000. At least 5.96 GB of clean data with > 92.16% of them above Q30 were produced for each sample. Reads were aligned using HISAT2 25 and transcripts assembled with StringTie 26. Differential expression analysis was performed with DESeq2 (fold change ≥ 1.5, FDR ≤ 0.05) 27. Raw data are available under NCBI BioProject PRJNA1303229 and SRA accession SRP607481.

Psoriasis skin RNA data from multiple datasets were downloaded from the GEO database and analyzed using R4.3.1 (https://cran.r-project.org/). Human data reanalysis: For human skin samples, we reanalyzed cell-type-specific* Csf1r* expression from the scRNA-seq dataset (GSE220116, 10 psoriasis patients/PS and 10 healthy normal controls/HC) and its relation to MPS and cytokine expression. mRNA expression differences in *Il34*, *Csf1* and *Ppara* were analyzed in two bulk RNA-seq databases: GSE13355 (PS/PN: 58 lesional/non-lesional skin samples from psoriasis patients and HC: 64 healthy normal skin samples) and GSE142582 (PS/PN: 5 lesional/perilesional skin samples from psoriasis patients and HC: 5 healthy normal skin samples). Murine Data Reanalysis: For mice skin samples, we integrated two scRNA-seq datasets (GSE230513: 2 healthy normal controls/CON and GSE231728: 2 IMQ-induced psoriasis mice/IMQ) and performed deconvolution analysis with our bulk RNA-seq data as described. 28 Plots were generated using Seurat (5.0.1) and scCustomize (1.1.3) R package.

### IHC

Human skin biopsies and murine dorsal skin were fixed in 10% formalin (24 h), paraffin-embedded, and sectioned (3 μm). Antigen retrieval: EDTA buffer (pH 9.0, 95 °C, 20 min). Blocking: 5% donkey serum. Primary antibodies were incubated at 4 °C for ≥ 18 h: rabbit anti-CSF1R (sc-692, Santa Cruz; 1:500), mouse anti-PPARα (66826-1, SAB, 1:500). Signal detection was carried out using ABC-HRP/DAB kits (Vector Labs), and sections were counterstained with hematoxylin. Images were captured using a KF-PRO-120-HI scanner at 10×/80× magnification. Positive^⁺^ cells in epidermis and/or dermis were quantified in 3 random non-overlapping fields per section (10×/80x objective) using the ImageJ Cell Counter plugin (n = 8-12 cases/group, 2-3 sections/case).

### IF

Human skin biopsies and murine dorsal skin samples were fixed in 10% neutral-buffered formalin for 24 h at room temperature (RT). Mouse skin samples were post-fixed in PFA solution for 3 h at 4 °C, and then cryoprotected in 30% sucrose for 72 h. After the tissues sank, they were embedded in OCT compound and cut into 20 µm coronal sections on a Leica CM3050 S cryostat. Free-floating sections were rinsed three times in TBS (0.01 M, pH 7.4) and blocked for 1 h at room temperature in TBS containing 5% normal donkey serum and 0.3% Triton X-100 (Sigma-Aldrich, T9284). Sections were incubated overnight at 4 °C with primary antibodies diluted in 5% serum-TBS. IF staining was performed by using rabbit anti-CSF1R (sc-692, Santa Cruz; 1:500), goat-anti-CSF1 (AF416, R&D System; 1:400), ribbit anti-Ki67 (ab16667, Abcam; 1:500), rat anti-F4/80 (123102, BioLegend, 1:100), mouse anti-CD3 (sc-20047, Santa Cruz, 1:100), and rat anti-NIMP-R14 (sc-59338, Santa Cruz, 1:). Following three washes, corresponding Alexa Fluor 488-, 555- or 647-conjugated secondary antibodies (1:500; Invitrogen) were applied for 1 h at RT in the dark. Finally, slices were mounted on slides with DAPI (SouthernBiotech, 0100-20). Images were captured with an EVOS FL Auto imaging system (Thermo Fisher) and high-resolution confocal stacks were acquired on a Nikon C2 microscope using identical laser power, pinhole size, and gain settings across all experimental groups. Analyses were performed by using ImageJ (NIH, v1.53). Values of integrated density (IntDen) from the control (CON or HC) group were normalized to 1, and data from remaining groups were expressed relative to this baseline. For each animal, 3-4 non-adjacent sections were randomly selected, and three fields per section were quantified (n = 3 mice per group). Results are presented as mean ± SEM.

### Flow cytometry

Single-cell suspensions were prepared from: Blow cytometry analysis was conducted on the skin and blood of mice. Peripheral blood was collected via cardiac puncture into EDTA tubes after anesthesia, followed by RBC lysis (10× RBC Lysis Buffer, BioLegend #420301; 10 min, RT). After transcardial PBS perfusion, skin tissues were minced and digested in Liberase (300 μg/mL) + DNase I (50 U/mL) for 60 min at 37 °C with agitation. Reactions stopped with 10% FBS-RPMI1640, filtered (70 μm), and centrifuged (450 ×g, 5 min, 4 °C). Staining protocol, Acquisition & Analysis: Zombie BV510 (1:400, 15 min, RT) was used for viability staining, α-CD16/32 (clone 2.4G2, 1:500, 10 min, RT) + True-Stain Monocyte Blocker (1:20) for Fc block. Cells were directly incubated with surface marker's antibodies (Table S3) in FACS buffer (PBS + 5% FBS) for 30 min at 4 °C or fixed and permeabilized with Cyto-Fast Fix/Perm (20 min, RT), then cytokines/Ki67 stained in Perm Wash buffer (20 min, 4 °C) for cytokine intracellular staining. Finally, cells were resuspended in 300 μL FACS buffer, filtered through a 70 μm cell strainer, and acquired on Cytek Aurora (5-laser configuration). ≥ 20,000 live CD45^⁺^ events/sample were collected and data were analyzed using FlowJo (V10.8.1).

### Subset definitions

During the process of flow cytometry analysis, live single cells were gated based on forward scatter area (FSC-A) and side scatter area (SSC-A) to isolate single live cells. Then according to these references, skin CD45^+^ cells were selected, and further gated subsets (Figure 2A- B): monocytes (Mo, CD45^+^CD11b^+^Ly6C^+^MHCII^-^EpCAM^-^F4/80^-^), macrophages (MΦ, CD45^+^CD11b^+^F4/80^+^CD64^+^); conventional DCs (cDCs, CD45^+^ CD11b^+^CD64^-^Ly6C^-^); and Langerhans cells (LCs, CD45^+^CD11b^+^EpCAM^+^F4/80^-^); monocyte-derived dendritic cells D1 (moDCs D1, CD45^+^CD11b^+^Ly6C^+^MHCII^+^ EpCAM^-^F4/80^-^); and moDCs D2 (CD45^+^CD11b^+^ Ly6C^-^MHCII^+^EpCAM^-^F4/80^-^). Flow cytometry gating strategy for immune cell analysis in mouse blood is based on the previous study 29-32. After isolate single live cells and CD45^+^ cells were selected from the blood, further gating was to identify (Figure S3C-D): neutrophils (CD45*^+^*CD11b^+^Ly6G^+^); CD11b^+^Ly6G^-^ Compartment (encompassing monocytes (Mo, CD45*^+^*^-^Ly6G^-^CD11b^+^CD11c^+^MHC-II^-^) and cDCs (CD45*^+^*Ly6G^-^CD11b^+^CD11c^+^MHC-II^+^); T cells (CD45*^+^*CD11b^-^Ly6G^-^CD3^+^).

### Pharmacological interventions

To investigate the pro-inflammatory effect of CSF1R activation, recombinant mouse CSF1 (rCSF1, RP01216, Abclonal; 0.5 μg/mouse) or IL-34 (rIL-34, YB031-5MP, UBI; 0.5 μg/mouse) was mixed with 0.1% BSA in sterile saline and injected subcutaneously in four back areas (total 100 μL) 30 min before daily low-dose IMQ (L-IMQ) treatment. For CSF1R inhibition, PLX3397 (S7818, Selleckchem; 0.1 or 1 mM in 100 μL vehicle) was administered subcutaneously 30 min before daily standard IMQ for 5 consecutive days. PPARα activity was modulated as follows: 1) The PPARα antagonist GW6471 (S2798, Selleck; 10 mM/100 μL) was subcutaneously injected from the day 1 to day 5 of IMQ treatment. 2) The PPARα agonist WY-14643 (S8029, Selleck; 10 mM/100 μL) was injected from day 2 to day 5 of IMQ treatment. These three compounds were dissolved in a carrier solution (5% DMSO, 40% PEG300, 5% Tween80, and 50% ddH_2_O), and the saline/vehicle control matched the administration schedule. All the compounds used in this study and the rationale for their selected doses are summarized in Table S4.

### *In vitro* macrophage assays

Resident peritoneal macrophages were used to model the CSF1R⁺ tissue-resident MPS hub identified in psoriatic skin, as they represent fully mature, self-renewing, embryonically seeded macrophages that retain *in vivo*-imprinted transcriptional programs and intact macrophage-specific enhancer landscapes 33, 34. Peritoneal macrophages were harvested by lavage with 10 mL ice-cold PBS 35, centrifuged (300 ×g, 10 min, 4 °C), resuspended in RPMI-1640 supplemented with 10% FBS, and seeded at 3×10⁵ cells/well (24-well plates). After 3 h adhesion, non-adherent cells were removed; flow cytometry confirmed a macrophage purity consistently > 90% (F4/80⁺CD11b⁺). Adherent macrophages were serum-starved overnight prior to stimulation. Cells were treated with: IMQ (10 μg/mL) ± PLX3397 (0.5 μM, 2 h pretreatment); low-dose IMQ (0.5 μg/mL) ± rCSF1 or rIL-34 (50 ng/mL) or IMQ + PLX3397 (0.5 μM) ± the PPARα antagonist GW6471 (5 μM, 2 h pretreatment). After 24 h, cells were rinsed and lysed for total RNA extraction and PCR analysis. The rationale and usage details for all concentrations applied in the *in vivo* and *in vitro* experiments are comprehensively summarized in Table S4.

### Quantitative reverse transcription polymerase chain reaction (qRT-PCR)

Total RNA was extracted from mouse back skin tissues or cultured peritoneal macrophages using the RNA Quick Purification kit (RN001, EScience) and reverse-transcribed into cDNA using ABScript III RT Master Mix (RK20429, Abclonal). qRT-PCR was performed with CFX 96 Touch (Bio-Bad) using 2× Universal SYBR Green Fast qPCR Mix (RK21203, Abclonal). Results were normalized to *Gapdh* expression. Primers are listed in Table S5.

### WB

Human samples: Skin samples were obtained from psoriasis patients at the dermatology clinic and from normal controls. Mouse samples: Skin (n = 3~4 mice/group) tissues were harvested after perfusing the animals transcardially with ice-cold PBS under urethane anesthesia. The skin was then homogenized in RIPA buffer containing 1 mM PMSF, followed by centrifugation (12,000 ×g for 15 min at 4 °C). Protein concentration was determined by BCA assay, and lysates (15 μg) were separated on 10% SDS-PAGE gels, transferred to 0.2-μm PVDF membranes, and blocked with 5% BSA/TBST. The membranes were incubated overnight at 4 °C with the following primary antibodies: rabbit anti-CSF1R (sc-692, Santa Cruz, 1:1000), goat anti-CSF1 (AF416, R&D System, 1:500), rabbit anti-IL-17A (66148-1-Ig, Proteintech, 1:1000), rabbit anti-β-actin (811161-RR, Proteintech,1:50,000), rabbit anti IL-34 (Invitrogen, PA5-95624, 1:1000), rabbit anti-IBA1 (019-19741, Wako, 1:1000), rabbit anti-IL-1β (ab9722, Abcam, 1:1000). After incubation with HRP-conjugated secondary antibodies (1:10,000, 1 h at RT), the signals were detected by ECL and imaged on a TANON-5200 imaging system. The relative optical density of each immunoreactive protein was quantified in ImageJ, with normalization to β-actin and the HC or CON group was the baseline.

### Statistical analysis

**Figure 1:**
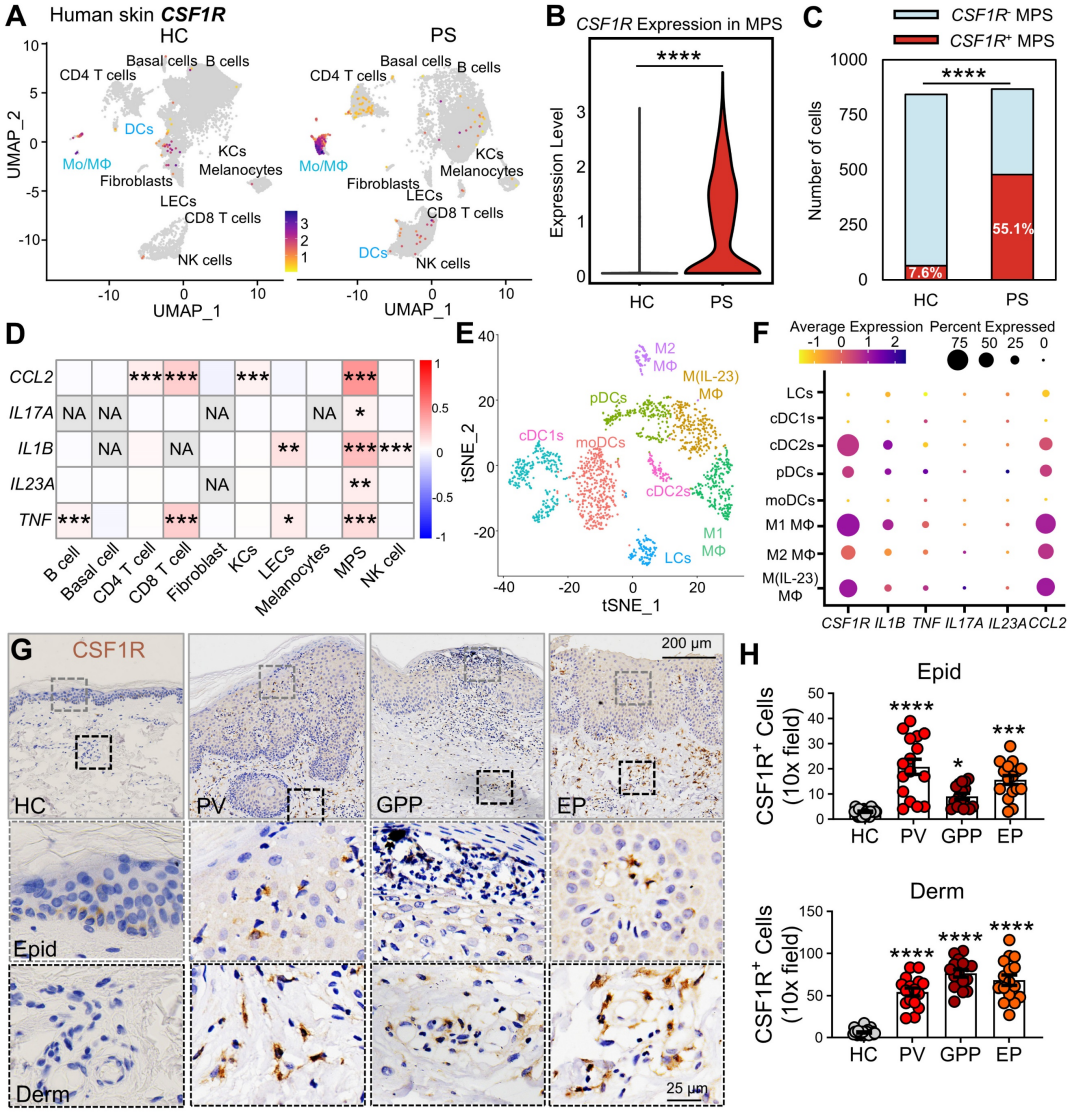
** CSF1R^+^ MPS subsets drive pro-inflammatory responses in human psoriasis skin. (A)** UMAP visualization of single-cell transcriptomes (scRNA-seq) from psoriatic (PS) versus (*vs.*) healthy (HC) skin. Color intensity represents the levels of *CSF1R* expression.** (B)** MPS cell-specific *CSF1R* expression in PS and HC cohorts. **(C)** Number of *CSF1R*^+^ and *CSF1R*^-^ cells among two groups. **(D)** Heatmap showing Spearman correlation coefficients between sample-level cell type enrichment scores and tissue-level cytokine gene expression derived from scRNA-seq data across human skin samples. Red indicates a significant positive correlation (P < 0.05), reflecting the co-occurrence of specific cell populations with cytokine-enriched inflammatory microenvironments, rather than cytokine expression within those cell types.** (E)** tSNE plot annotating MPS subclusters in PS skin. **(F)** Dot plot showing co-expression of *CSF1R* with psoriasis-associated cytokines (*TNF*, *IL1B*, *IL17A*, *IL23A*, *CCL2*) in MPS subclusters. Circle size represents expression prevalence, and color indicates the average expression level. **(G, H)** Immunohistochemical detection of CSF1R^+^ cells in epidermis (Epid) and dermis (Derm). Quantification includes 8-12 biopsies/group (2-3 sections/biopsy). Data are presented as mean ± SEM; each point in the scatter plots represents a sample. P values: *P < 0.05, **P < 0.01, ***P < 0.001, ****P < 0.0001 *vs.* HC group.

Data are presented as mean ± SEM, with individual data points shown in scatter plots. All analyses were performed using GraphPad Prism 8 (v8.4.3). Skin fold thickness or PASI scores were analyzed using two-way ANOVA, followed by either Bonferroni's or Tukey's multiple comparison test, depending on the specific comparison. Multi-group comparisons were initially performed using ordinary one-way ANOVA. The Chi-square test was applied to examine the proportional differences in cell types between groups (Figure 1C). A two-tailed unpaired t-test or Mann-Whitney test were used to compare two groups. Spearman's rank correlation was employed for correlation analyses. For RNA-seq differential expression analysis (DEGs), DESeq2 was used with FDR-adjusted P-values. Statistical significance at P < 0.05.

### Study approval

The human study protocol was approved by the Institutional Review Board (IRB [2022] 503) in accordance with the Declaration of Helsinki, and all participants had provided written informed consent after their clinical data had been anonymized with unique identification codes. Animal procedures were carried out in compliance with ARRIVE guidelines and were approved by the Institutional Animal Care and Use Committee (IACUC) of Sun Yat-sen University (SYXK(yue)2017-0084; 2022-0081).

## Results

### Pathogenic MPS hubs exhibit compartmentalized CSF1R overexpression in psoriatic lesions

Given the emerging role of CSF1R as a pan-MPS marker and its therapeutic potential in inflammatory diseases 10, 22, 36, we first assessed its expression in human psoriatic lesions. Reanalysis of human skin single-cell RNA-sequencing (scRNA-seq) data [GSE220116] 37 revealed a significant expansion of MPS cells (Mo/MΦ and DCs) in psoriatic (PS) lesions compared to healthy control (HC) skin. MPS cells were annotated with standard markers (Figure S1A-B), and this expanded MPS population exhibited markedly elevated CSF1R expression (Figure 1A-B). CSF1R⁺ MPS cells were substantially increased in PS lesions (Figure 1C). To assess the relationship between cellular composition and inflammatory signaling at the tissue level, we performed Spearman correlation analysis between cell-type enrichment scores and bulk cytokine expression profiles. This analysis demonstrated that enrichment of CSF1R⁺ MPS subsets was strongly and positively correlated with key psoriasis-associated cytokines, including *IL1B*, *TNF*, *IL23A*, and *IL17A* (Figure 1D). In contrast, enrichment of bulk CD4⁺ or CD8⁺ T-cell populations exhibited weaker or non-significant correlations with *IL17A* expression. Importantly, this analysis reflects tissue-level co-occurrence rather than gene expression within individual cell types, indicating that samples enriched for CSF1R⁺ MPS cells are more likely to display IL-17-dominated inflammatory signatures. Subclustering analysis identified the highest CSF1R expression in pro-inflammatory M1 (*IL1B*⁺) and M(*IL-23*) (*IL17A⁺IL23A⁺*) macrophages, followed by cDC2s and plasmacytoid dendritic cells (pDCs), while minimal expression was observed in M2 macrophages, LCs, cDC1s, and monocyte-derived dendritic cells (moDCs) (Figure S1C, Figure 1E-F). These CSF1R^high^ pro-inflammatory MPS subsets showed enhanced co-localization with *IL1B*, *TNF*, *IL17A*, *IL23A*, and *CCL2* in DCs and LCs, suggesting that CSF1R^high^ MPS cells form localized cytokine amplification hubs.

Immunohistochemical analysis across psoriasis subtypes confirmed substantial accumulation of CSF1R^high^ cells in both epidermal and dermal compartments across psoriasis subtypes compared to HC skin (Figure 1G-H). Epidermal CSF1R^+^ cells increased 6.7-fold in PV, 2.9-fold in GPP, and 5.0-fold in EP lesions, while dermal increases reached 9.5-, 12.4-, and 10.3-fold, respectively. These findings implicate CSF1R^high^ MPS cells in driving psoriasiform inflammation through keratinocyte crosstalk.

**Figure 2:**
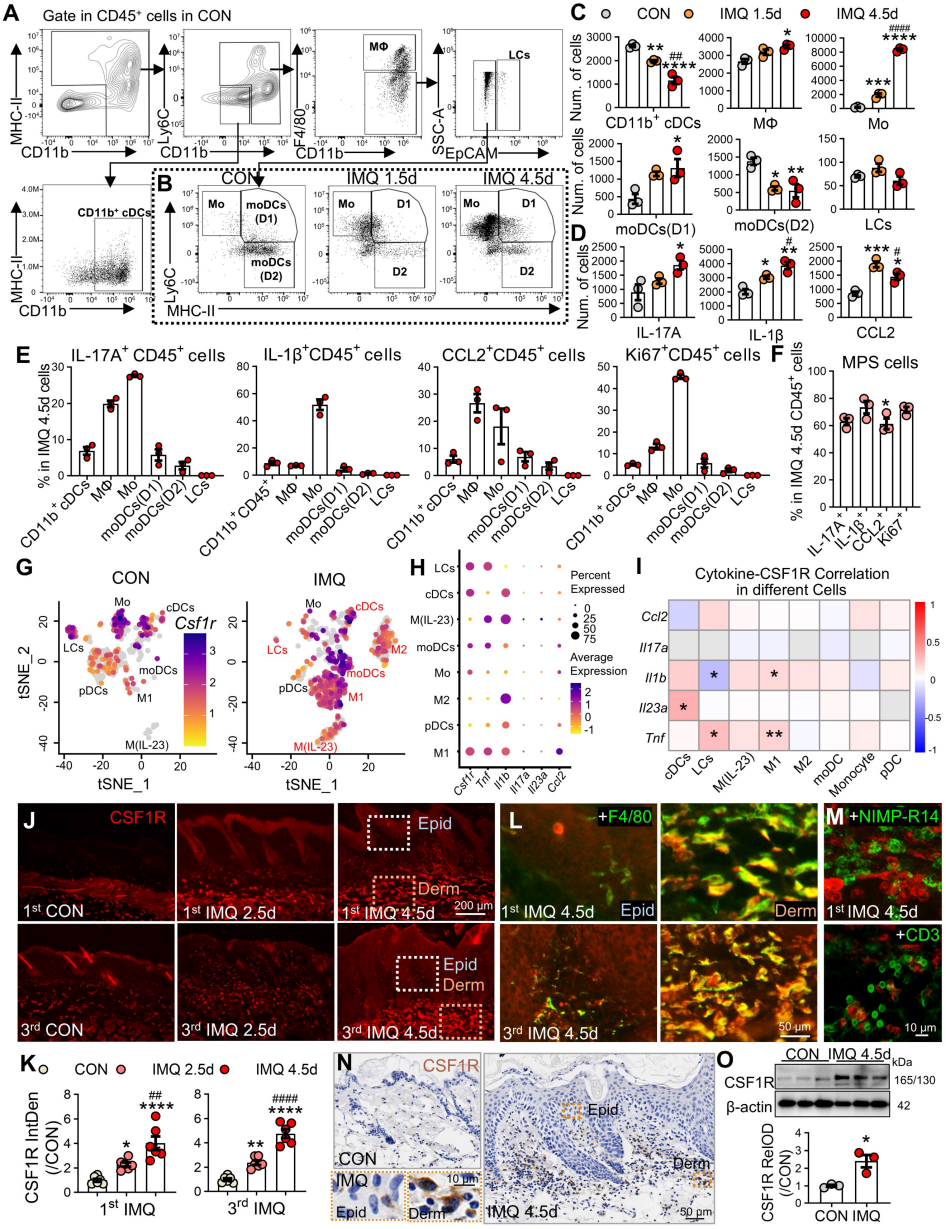
** Spatiotemporal expansion of pathogenic CSF1R⁺ MPS subsets in murine psoriatic lesions. (A, B)** Gating strategy for skin immune cells and representative flow cytometry plots comparing different groups.** (C, D)** Quantification of MPS subsets and cytokine⁺ cells (IL-17^+^, IL-1β^+^, CCL2^+^) among 2 × 10^4^ CD45^+^ cells across different groups (n = 3/group). **(E, F)** Functional profiling at IMQ 4.5d: (E) Percentage of cytokine⁺ cells within specific MPS subsets; (F) Percentage of total cytokine⁺/Ki67⁺ MPS cells in cytokine^+^/Ki67^+^CD45^+^ cells.** (G)** tSNE embedding of MPS subclusters in CON and IMQ samples from murine scRNA-seq data (GSE230513/ GSE231728).** (H)** Dot plot summarizing percent-expressing fraction and average expression of Csf1r and psoriasis-associated cytokine genes (*Il1b*, *Tnf*, *Il17a*, *Il23a*, *Ccl2*) across MPS subtypes. **(I)** Heatmap of Spearman correlation coefficients between* Csf1r* and cytokine genes within MPS subtypes. *P < 0.05, **P < 0.01 denote correlations significant after Benjamini-Hochberg FDR correction (fold change ≥ 1.5, FDR ≤ 0.05). **(J-M)** Representative IF images and quantification (J, K) of CSF1R protein dynamics during the initial (1^st^ IMQ) and recurrent (3^rd^ IMQ) phases of psoriasis. Day 2.5 was selected to compare the intensity of inflammation at the intermediate phase, highlighting the rapid recall response in the recurrent model. Co-localization of CSF1R⁺ (red) with F4/80^+^ macrophages (green) (L), NIMP-R14^+^ neutrophils or CD3^+^ T cells (M). **(N, O)** Validation of CSF1R expression at IMQ 4.5d using IHC (N) and WB (O). *P < 0.05, **P < 0.01, ***P < 0.001, ****P < 0.0001 *vs.* CON group; ^#^P < 0.05, ^##^P < 0.01, ^####^P < 0.0001 *vs.* IMQ 2.5d group.

In the IMQ-induced murine model, flow cytometry revealed dominant expansion of macrophages and monocytes during disease progression (Figure 2A-C). Monocytes increased 10.9-fold at day 1.5 and 48.1-fold at day 4.5 post-IMQ, while macrophages showed a 1.3-fold expansion by day 4.5. Cytokine profiling demonstrated progressive increases in IL-17⁺ and IL-1β⁺ CD45⁺ cells, with CCL2⁺ cells peaking early at day 1.5 (Figure 2D). Cellular source analysis identified monocytes as the primary producers of IL-17 (27.6%) and IL-1β (51.9%), while macrophages dominated CCL2 production (Figure 2E). Proliferation analysis revealed active expansion of pro-inflammatory MPS subsets, with 45.4% of monocytes and 13.3% of macrophages being Ki67⁺ at day 4.5 (P < 0.001). Overall, 60-70% of cytokine-positive CD45⁺ cells at day 4.5 were derived from the MPS compartment (Figure 2F).

Murine scRNA-seq reanalysis (GSE230513, GSE231728) corroborated these findings, showing MPS cells constituted 81.4% of expanded immune cells in IMQ-treated skin (P < 0.0001; Figure S2A-C). *Csf1r* expression was selectively upregulated in MPS cells following IMQ treatment, paralleling increased expression of *Il1b*, *Tnf*, and *Ccl2* (Figure S2D). Subclustering analysis revealed enrichment in moDCs, M1, and M(IL-23) subsets that co-expressed pathogenic cytokines (Figure S2E, Figure 2G-H). Joint-density visualization confirmed *Csf1r* co-localization with inflammatory mediators in M1 cells (Fig S2F), and Spearman correlation analysis showed significant positive correlations between *Csf1r* and *Il1b*, *Tnf,* or *Il23a* in pro-inflammatory subsets (Figure 2I). Immunofluorescence validation demonstrated significant CSF1R protein upregulation during both initial disease induction and recurrent phases (2.3-4.8-fold; P < 0.0001; Figure 2J-K). CSF1R immunoreactivity specifically co-localized with F4/80⁺ macrophages (Figure 2L), but not neutrophils or T cells (Figure 2M). WB and immunohistochemical analyses further confirmed CSF1R protein elevation in lesional skin (Figure 2N-O).

### Circulating CSF1R⁺ monocytes amplify systemic inflammation

**Figure 3:**
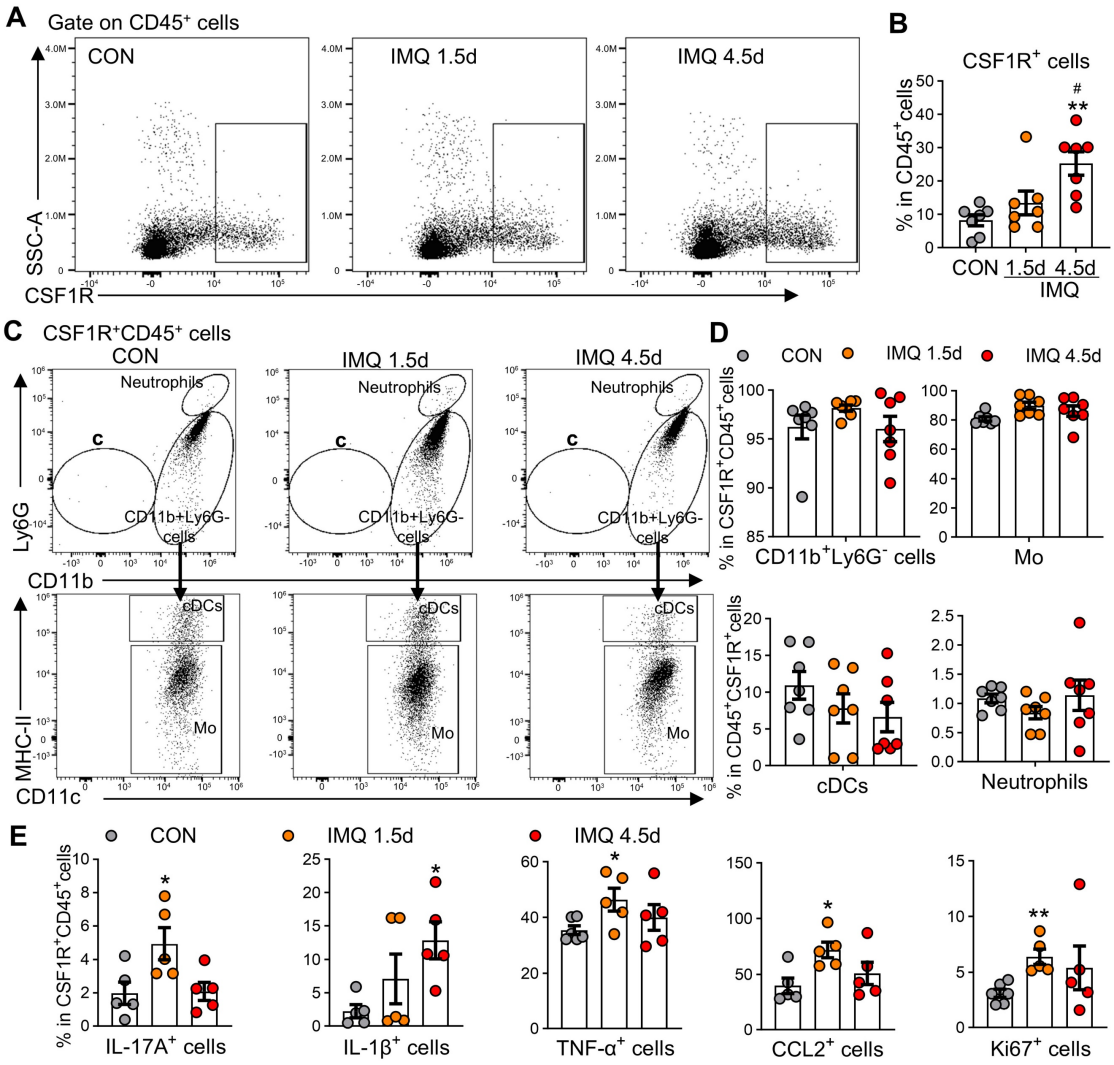
** Expansion of CSF1R^+^ cells in peripheral blood of IMQ-treated mice. (A, B)** Representative flow cytometry dot plots (A) and quantitative analysis (B) showing the proportion of CSF1R^+^ cells among CD45^+^ cells in mouse blood at different IMQ treatment time points (CON, IMQ 1.5d, IMQ 4.5d).** (C, D)** Distribution and proportion (%) of CSF1R^+^CD45^+^ cells within immune cell subsets, including CD11b^+^Ly6G^-^ cells, Mo, cDCs, and neutrophils. **(E)** Percentage of IL-17A^+^, IL-1β^+^, TNF-α^+^, CCL2^+^, or Ki67^+^ cells within the CSF1R^+^CD45^+^ population at IMQ 1.5d and IMQ 4.5d. Data from 3-6 mice/group, *P < 0.05, **P < 0.01 *vs.* CON; ^#^P < 0.05 *vs.* IMQ 1.5d.

Psoriasis involves dysregulated interactions between cutaneous and systemic immunity,^38^ with peripheral MPS alterations contributing to disease pathogenesis 39, 40. Patients with PV and EP subtypes exhibited significant monocytosis (PV: 1.4-fold increase; EP: 1.3-fold increase vs. HC, P < 0.01; Figure S3A) and elevated monocyte-to-lymphocyte ratios (Figure S3B), a recognized biomarker of systemic inflammation 41. Mirroring human findings, IMQ-treated mice showed time-dependent expansion of CD11b⁺Ly6G⁻ cells (encompassing monocytes/myeloid-derived suppressor cells) with a 1.8-fold increase by day 4.5 (P < 0.001), while neutrophils (CD11b⁺Ly6G⁺) transiently peaked at day 1.5 (2.3-fold increase; P < 0.05; Figure S3C-E). Flow cytometry revealed progressive expansion of CSF1R⁺CD45⁺ cells (3.1-fold by day 4.5 post-IMQ, P < 0.01; Figure 3A-B). These CSF1R⁺CD45⁺ cells were predominantly localized to CD11b⁺Ly6G⁻ subsets (96.5%), with monocytes constituting the vast majority (86-89%; Figure 3C-D). Functional characterization demonstrated temporal increases in cytokine-producing cells within this population: IL-17A⁺ (2.5-fold), IL-1β⁺ (5.7-fold), TNF-α⁺ (1.3-fold), and CCL2⁺ (1.7-fold) cells (all P < 0.05; Figure 3E). Enhanced proliferative capacity was evidenced by a 2.0-fold increase in Ki67⁺ cells at day 1.5 (P < 0.01). These results identify circulating CSF1R⁺CD45⁺ MPS cells as peripheral amplifiers of psoriasis through hypersecretion of inflammatory cytokines.

### Spatiotemporal dysregulation of CSF1R ligands fuels pathogenic circuits in psoriasis

**Figure 4:**
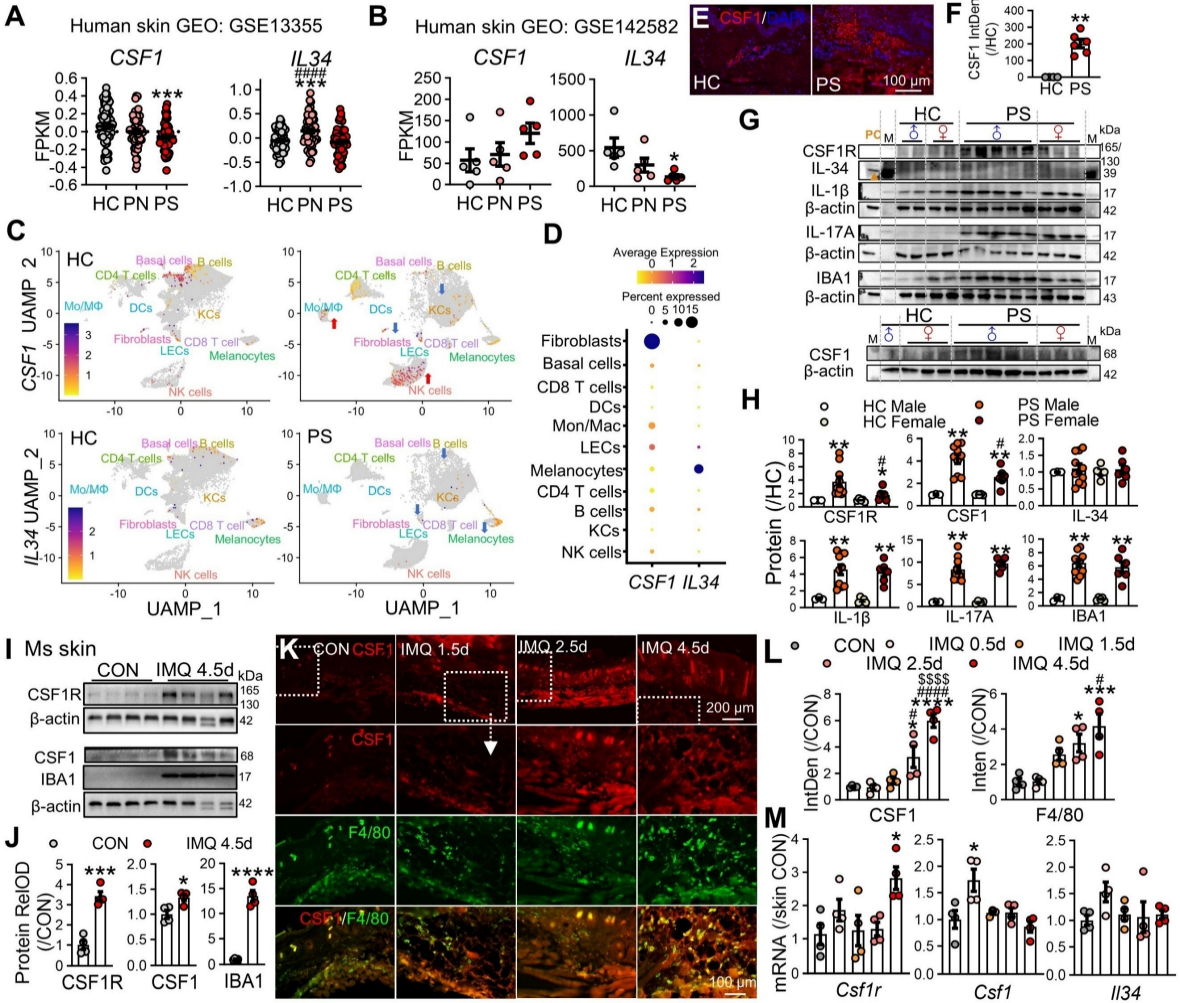
** Spatiotemporal dysregulation of CSF1R ligands in psoriasis. (A, B)** Transcriptomic analysis of *CSF1* and *IL34* expression in human psoriasis datasets GSE13355 (A) and GSE142582 (B). PN: uninvolved skin from psoriasis patients; PS: lesional skin from psoriasis patients. **(C, D)** scRNA-seq analysis (GSE220116) visualizing *CSF1* and *IL34* expression across major skin cell populations in PS and HC samples.** (E, F)** Representative IF images and quantification of CSF1 expression in lesional psoriatic skin from male and female PS and HC samples.** (G, H)** Representative WB bands and quantitative analysis of CSF1R pathway-related proteins in human skin samples from PS and HC, stratified by sex (male ♂, female ♀). HaCaT cell lysates were used as a positive control for IL-34 (PC, indicated by brown triangle). M: molecular weight markers. **(I, J)** WB and quantitative analysis of CSF1R, Iba1, and CSF1 protein expression in mouse skin at IMQ 4.5d. **(K, L)** IF imaging and quantitative analysis of CSF1 (red) and F4/80 (green) co-localization in mouse skin. **(M)** qRT-PCR results showing temporal dynamics of *Csf1r*, *Csf1*, and *Il34* mRNA expression during IMQ-induced psoriasiform inflammation. *P < 0.05, **P < 0.01, ***P < 0.001, ****P < 0.0001 *vs.* HC (male/female) or CON group; ^#^P < 0.05 *vs.* PS or IMQ 0.5d; ^$^P < 0.05 *vs.* IMQ 1.5d.

To characterize CSF1R ligand regulation in psoriasis, we integrated human transcriptomic datasets with protein-level and functional analyses. Bulk RNA-seq of human psoriatic skin (GSE13355) revealed spatially distinct ligand expression, with reduced* CSF1* in lesional cores (PS) and elevated *IL34* in perilesional regions (PN) (P < 0.001; Figure 4A). Analysis of an independent cohort (GSE142582) confirmed altered intralesional ligand patterns, showing decreased IL34 and a trend toward increased CSF1 in lesional skin (Figure 4B).

Reanalysis of scRNA-seq data (GSE220116) demonstrated a disease-associated shift in ligand cellular sources. In healthy skin, *CSF1* was primarily expressed by fibroblasts and B cells, whereas in psoriatic lesions *CSF1* expression was enriched in inflammatory monocytes/macrophages and NK cells. In contrast,* IL34* expression remained low across multiple structural cell types, including keratinocytes, melanocytes, and lymphatic endothelial cells (Figure 4C-D).

At the protein level, immunofluorescence analysis revealed robust CSF1 expression in psoriatic lesions, with minimal signal in healthy control skin (Figure 4E-F). Sex-stratified WB analyses further demonstrated that CSF1R and CSF1, together with IL-1β, IL-17A, and the myeloid marker IBA1, were markedly upregulated in lesional skin from both sexes, with a significantly greater magnitude of induction observed in male patients, whereas IL-34 protein levels remained unchanged (Figure 4G-H). Consistently, sex-stratified scRNA-seq analysis revealed that overall MPS abundance was comparable between male and female patients (Figure S4A), while CSF1R expression levels within the pathogenic MPS subset were significantly higher in males (Figure S4B). Together, these data indicate that CSF1 upregulation represents a sex-independent feature of psoriatic lesions, whereas sex-associated differences emerge at the level of CSF1R expression intensity within pathogenic MPS compartments.

Consistent with human data, IMQ-treated mouse skin exhibited marked upregulation of CSF1R, CSF1, and Iba1 (Figure 4I-J), accompanied by the formation of dense CSF1⁺F4/80⁺ macrophage clusters in dermal cores (Figure 4K-L), indicative of organized autocrine signaling hubs. Temporal mRNA profiling revealed early induction of Csf1 (day 0.5), followed by delayed Csf1r upregulation (day 4.5), while Il34 expression remained relatively stable, suggesting phased pathway activation (Figure 4M).

**Figure 5:**
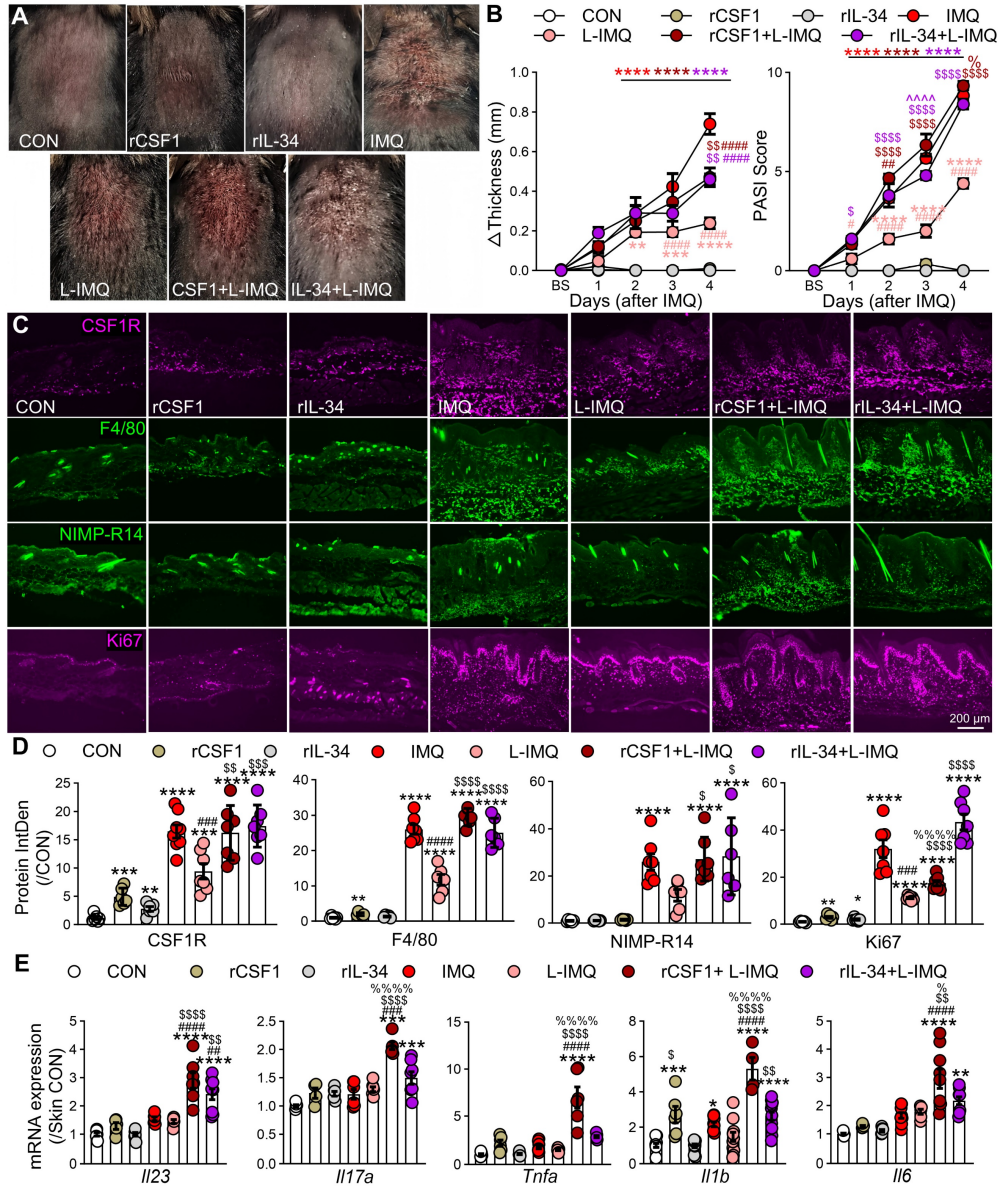
** Subcutaneous injection of CSF1R ligands exacerbates L-IMQ-induced dermatitis. (A)** Representative mouse skin appearance after 4.5 d treatment. Mice received subcutaneous injections of PBS (vehicle), recombinant mouse CSF1 or IL-34 (rCSF1 or rIL-34, 5 μg/mL, 25 μg/kg) 30 min prior to daily topical application of Vaseline (vehicle), standard-dose IMQ (62.5 mg/d), or low-dose IMQ (L-IMQ, 20.8 mg/d). **(B)** Changes in mouse skin fold thickness (left) and PASI scores (right) at different treatment time points (n = 3~6 mice/group). BS: baseline. **(C, D)** Representative IF images (C) and statistical quantification (D) of CSF1R, F4/80, NIMP-R14, and Ki67 signals in mouse skin at IMQ 4.5d (n = 3 mice/group, 2-3 slices/mouse).** (E)** mRNA expression levels of inflammatory cytokines (*Il23*, *Il17a*, *Tnfa*, *Il1b*, *Il6*) in skin lesions at day 2.5 (3 mice/group, 2-4 skin samples/mouse). *P < 0.05, **P < 0.01, ***P < 0.001, ****P < 0.0001 *vs*. CON; ^#^P < 0.05 *vs*. IMQ group, ^$^P < 0.05 *vs*. L-IMQ group; ^%^P < 0.05 *vs*. rIL-34 + L-IMQ group.

Functionally, subcutaneous administration of rCSF1 or rIL-34 (25 μg/kg) in combination with L-IMQ significantly exacerbated psoriasiform dermatitis, increasing skin thickness and clinical scores compared with IMQ alone (Figure 5A-B). Both the combination with ligands and L-IMQ enhanced CSF1R expression, F4/80⁺ macrophage and neutrophil infiltration and Ki67⁺ cell proliferation (Figure 5C-D), and even exceeded standard IMQ levels. Notably, rIL-34 + L-IMQ preferentially exacerbated epidermal hyperplasia and scaling, whereas rCSF1 + L-IMQ more strongly induced inflammatory cytokine transcripts, including *Il23a*, *Il17a*, *Tnfa*, *Il1b*, and* Il6 (*P < 0.05 vs. IL-34; Figure 5E). Collectively, these findings establish that spatiotemporal dysregulation of CSF1R ligands—particularly CSF1—drives pathogenic MPS activation, proliferation, and cytokine amplification through reinforced autocrine circuits in psoriasis.

### Systemic CSF1R targeting depletes pathogenic reservoirs across compartments

To evaluate therapeutic targeting of the CSF1R-MPS axis, we first assessed local CSF1R inhibition using subcutaneous PLX3397 (0.1 or 1 mM) administered 30 min before IMQ challenge (Figure S5A). PLX3397 dose-dependently reduced skin thickening and PASI, suppressed CSF1R, F4/80⁺ macrophage and NIMP-R14⁺ neutrophil infiltration, and Ki67⁺ proliferation (Figure S5B-E).

Mechanistically, PLX3397 downregulated key inflammatory cytokines (*Il23a*, *Il17a*, *Tnfa*, and *Il6*) in lesional skin (Figure S5F). *In vitro*, PLX3397 pretreatment (0.5 μM) blocked IMQ-induced overexpression of *Il17a*, *Tnfa*, and *Il6* in peritoneal macrophages without altering *Csf1* or *Il34* expression (Figure S5G), confirming direct CSF1R-mediated effects. These data demonstrate that local CSF1R blockade attenuates inflammation but fails to achieve complete resolution, implicating systemic MPS populations in disease maintenance.

**Figure 6:**
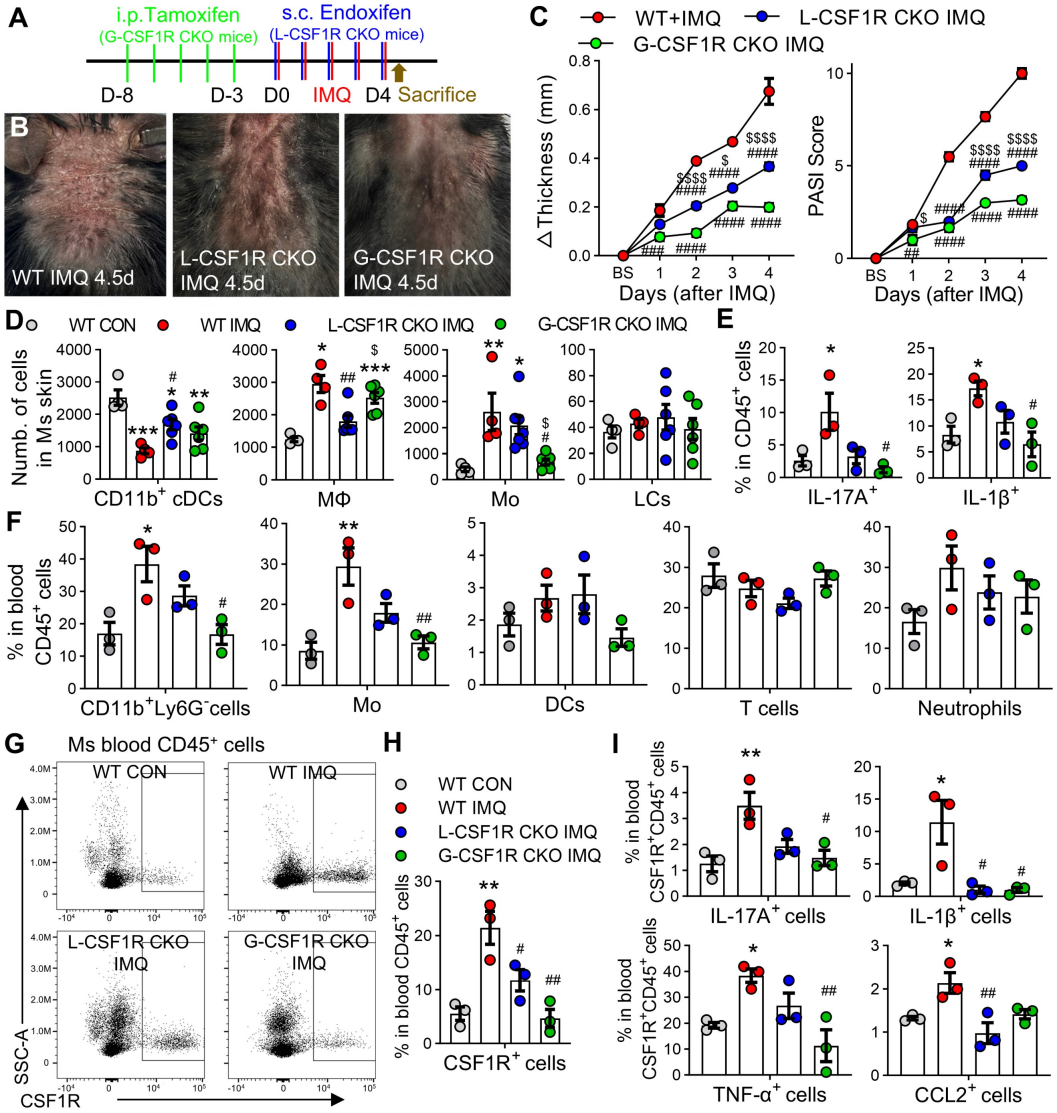
** Systemic CSF1R ablation more effectively reduces psoriasiform inflammation than local knockdown. (A)** Experimental schematic: *Cx3cr1*^CreER/+^; *Csf1rr*^fl/fl^ mice received global CSF1R conditional knockout (G-CSF1R CKO) via intraperitoneal injections of tamoxifen (Tam, 100 mg/kg/d, 5d) or local CSF1R conditional knockout (L-CSF1R CKO) via subcutaneous injections of endoxifen (Endox, 10 mg/kg/d, 5d). WT littermates received corn oil vehicle. All groups received topical IMQ (62.5 mg/d) for 4.5 days starting 24 h after the last injection. **(B)** Representative dorsal skin phenotypes at IMQ 4.5d.** (C)** Changes in skin thickness (left) and PASI scores (right) over time (n = 6 mice/group). **(D)** Flow cytometry quantification of MPS cell subsets (CD11b^+^ cDCs, MΦ, Mo, LCs) among skin CD45^+^ cells at day 4.5 (n = 4-6 mice/group). **(E)** Percentage of IL-17A^+^ and IL-1β^+^ cells among skin CD45^+^ cells (n = 3 mice/group). **(F)** Proportion of immune cell subsets (CD11b^+^Ly6G^-^ cells, Mo, cDCs, neutrophils, T cells) among peripheral blood CD45^+^ cells (n = 3 mice/group). **(G, H)** Representative flow cytometry plots (G) and quantification (H) of CSF1R^+^ cells among peripheral blood CD45^+^ cells. **(I)** Percentage of IL-17A^+^, IL-1β^+^, TNF-α^+^, or CCL2^+^ cells among peripheral blood CSF1R^+^CD45^+^ cells. *P < 0.05, **P < 0.01, ***P < 0.001 *vs.* WT CON; ^#^P < 0.05 *v*s. WT IMQ.

To directly compare systemic versus local CSF1R targeting, we employed *Cx3cr1*^CreER/+^; *Csf1r*^fl/fl^ mice with compartment-specific knockout. Global CSF1R knockout (G-CSF1R CKO) was induced by intraperitoneal injection of tamoxifen, while local knockout (L-CSF1R CKO) was achieved via subcutaneous endoxifen administration (Figure 6A). G-CSF1R CKO produced significantly greater reductions in skin thickness and clinical scores than L-CSF1R CKO (Figure 6B-C).

Flow cytometry revealed that L-CSF1R CKO primarily normalized CD11b⁺ conventional DCs (cDCs) and macrophages in skin, whereas G-CSF1R CKO more potently reduced monocyte accumulation (Figure 6D). Critically, G-CSF1R CKO disrupted cytokine networks by reducing IL-17A⁺ and IL-1β⁺ CD45⁺ skin cells (Figure 6E) and significantly depleted circulating CD11b⁺Ly6G⁻ cells, monocytes, and other immune subsets versus both WT IMQ and L-CSF1R CKO groups (Figure 6F). Furthermore, systemic ablation markedly reduced CSF1R⁺ cells among blood CD45⁺ cells (Figure 6G-H) and suppressed IL-17A, IL-1β, TNF-α, and CCL2 expression within this circulating population (Figure 6I). These results establish that systemic CSF1R targeting disrupts pathogenic networks more effectively than local blockade by depleting both tissue-infiltrating and circulating pro-inflammatory monocytes and suppressing cytokine production across compartments.

### CSF1R haploinsufficiency reprograms myeloid-keratinocyte crosstalk and ameliorates psoriasiform inflammation

**Figure 7:**
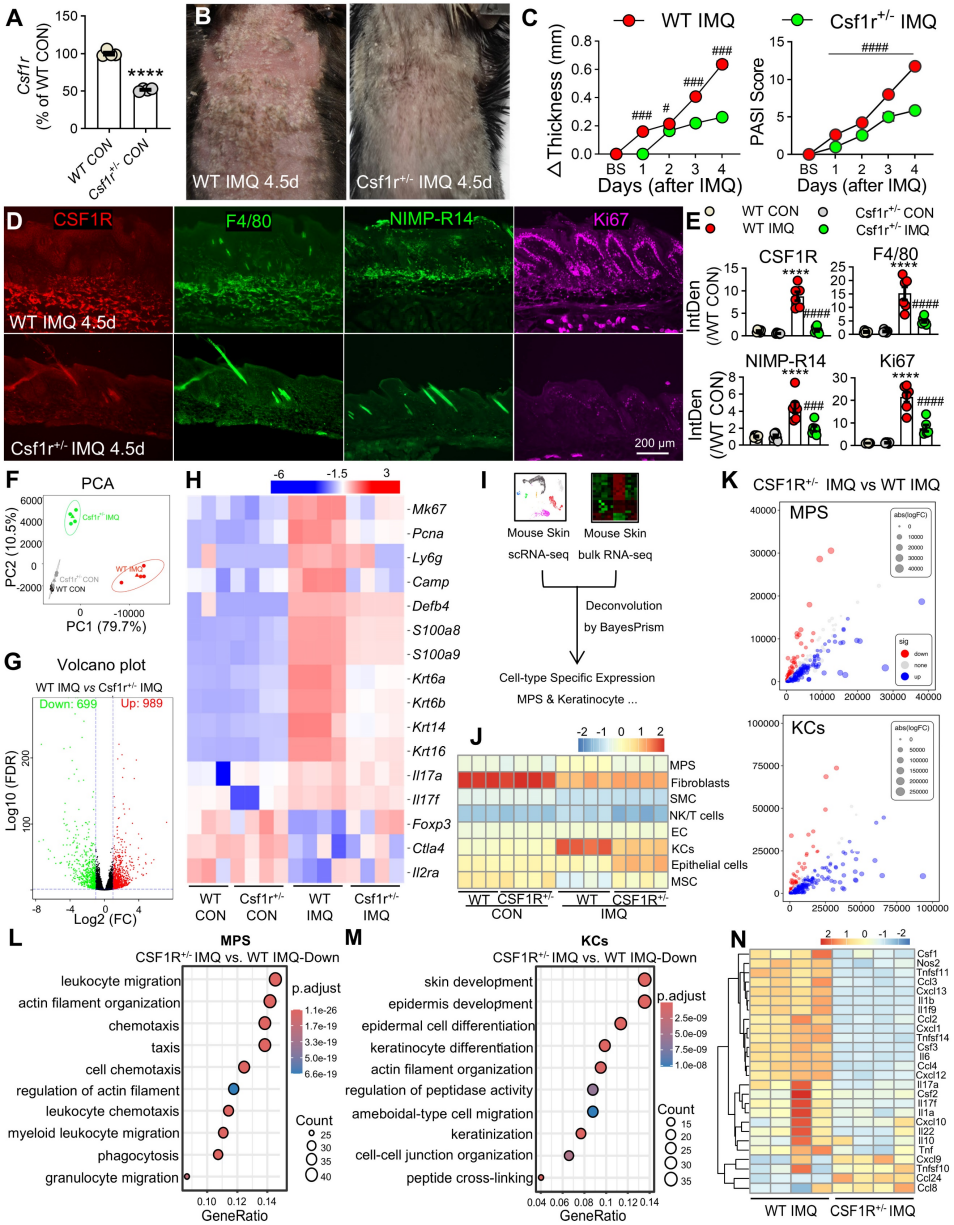
** CSF1R deficiency reprograms keratinocyte-myeloid crosstalk to resolve epidermal pathology. (A)** *Csf1r* mRNA reduction in *Csf1r*⁺/⁻ mice (n = 4 mice/ group). **(B, C)** Attenuated skin inflammation phenotypes at IMQ 4.5d (n = 7-8/group).** (D, E)** Reduced pathogenic infiltrates and keratinocyte hyperproliferation (IF quantification) (n = 3 mice/group; 2 sections/mouse). **(F)** PCA showing distinct transcriptional clustering (WT CON: n = 3; others: n = 4).** (G)** Volcano plot of DEGs (FC ≥ 1.5, FDR ≤ 5%). **(H)** Heatmap of key DEGs restored in* Csf1r⁺/⁻* mice. (**I**) Deconvolution workflow integrating RNA-seq (WT/*Csf1r*^+/-^ IMQ) with scRNA-Seq references (GSE230513 and GSE231728). **(J)** BayesPrism deconvolution revealing normalized reduced MPS infiltration and keratinocyte proportions. SMC: Smooth Muscle Cells, EC: Endothelial Cells, MSC: Mesenchymal Stem Cells. (**K**) Bubble plot of cell-type-specific DEGs.** (L, M)** GO-BP analysis showing top 300 upregulated DEGs in leukocyte migration (MPS) and skin development (keratinocytes). **(N)** MPS polarization shift from pro-inflammatory to regulatory phenotypes. *P < 0.05, **P < 0.01, ***P < 0.001 *vs*. WT CON; ^#^P < 0.05 *vs.* WT IMQ.

To investigate the functional role of CSF1R's role in psoriasis pathogenesis, we utilized *Csf1r*^+/-^ mice with validated reduced *Csf1r* expression (Figure 7A). Compared to wild-type (WT) controls, *Csf1r*^+/-^ mice exhibited significantly attenuated IMQ-induced skin thickening, clinical scores, and reduced infiltration of CSF1R⁺ cells, F4/80⁺ macrophages, neutrophils, and Ki67⁺ proliferating cells (Figure 7B-E). Bulk RNA-seq analysis of lesional skin identified 1,688 differentially expressed genes (DEGs) between Csf1r⁺/⁻ IMQ and WT IMQ groups (989 upregulated, 699 downregulated; FC ≥ 1.5, FDR ≤ 5%; Figure 7F-G). CSF1R haploinsufficiency suppressed the expression of proliferation markers (*Mki67*, *Pcna*), hyperproliferative keratin (*Krt6a*/*b*, *Krt14*, *Krt16*), neutrophil markers (*Ly6g*, *Camp*, *Defb4*, *S100a8*, *S100a9*) and pro-inflammatory cytokines (*Il23a*, *Il6, Il17a*/*f*), while restoring Treg markers (*Foxp3*, *Ctla4*, *Il2ra*) (Figure 7H).

Cellular deconvolution analysis revealed altered cellular distributions in Csf1r⁺/⁻ IMQ skin, with reduced MPS proportions and normalized keratinocyte populations (Figure 7I-K). Cell-type-specific DEG analysis highlighted divergent transcriptional programs: MPS cells showed enrichment for leukocyte migration and actin remodeling pathways (Figure 7L), while keratinocytes exhibited enhanced skin development and differentiation signatures (Figure 7M). Notably,* Csf1r*⁺^/-^ MPS cells displayed reduced pro-inflammatory signatures (M1: *Ccl8*, *Cxcl9*; M(IL-23): *Il17a/f*, *Tnfsf11/14*) and elevated M2 marker (*Ccl24*) (Figure 7N). These findings demonstrate that CSF1R deficiency restrains psoriatic inflammation by reprogramming MPS polarization and normalizing keratinocyte differentiation programs.

### CSF1R activation directly suppresses PPARα signaling in psoriatic microenvironments

**Figure 8:**
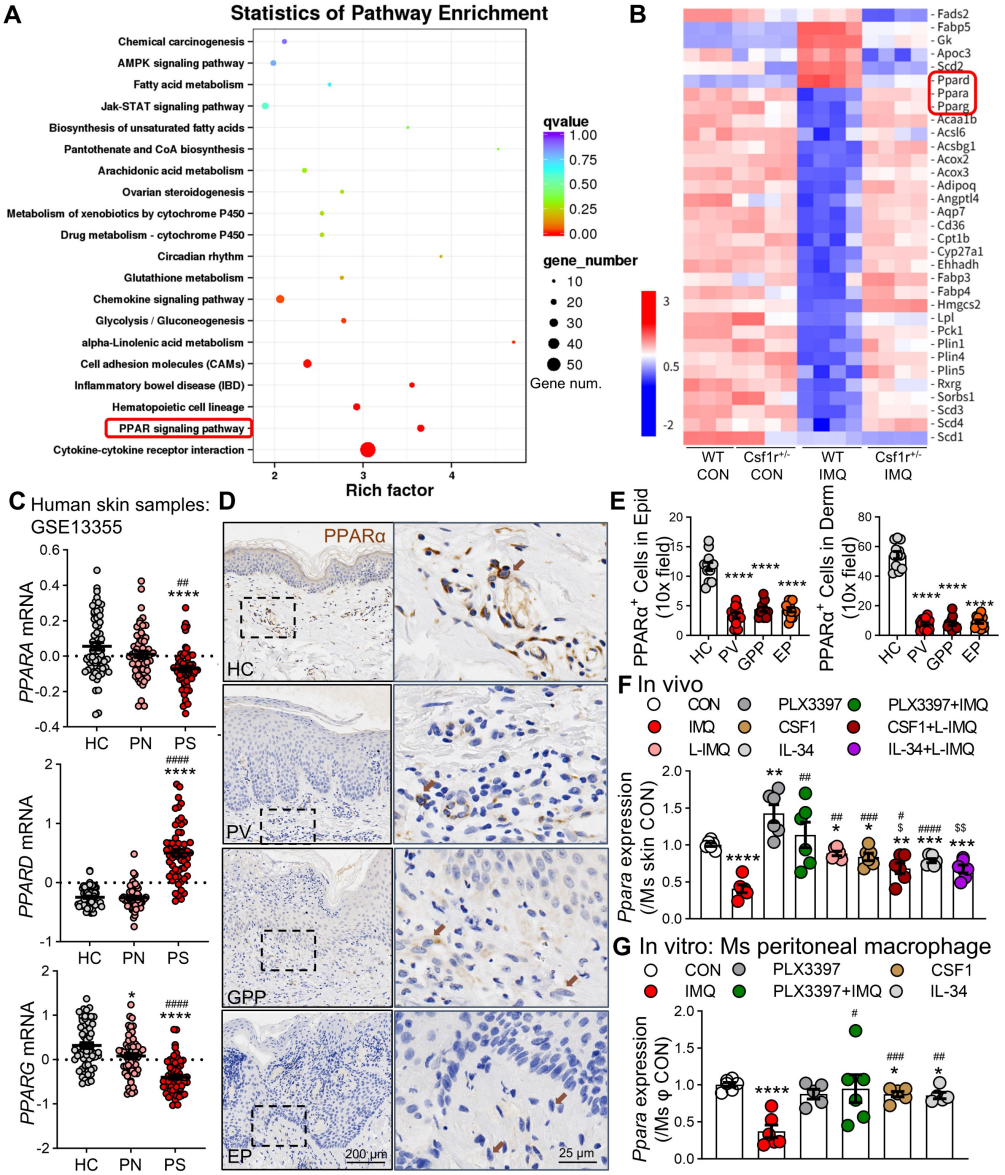
** PPARα is downregulated by CSF1R signaling in psoriatic skin and peritoneal macrophages. (A)** KEGG enrichment analysis of mouse skin DEGs (WT IMQ *vs*. *Csf1r^+/-^* IMQ) highlighting the PPAR pathway.** (B)** Heatmap of 33 DEGs in the PPAR pathway. **(C)** *PPARA, PPARD,* and* PPARG* expression in in human skin (GSE13355: HC n = 64, PN n = 58, PS n = 58). **(D, E)** IHC images (D) and quantification (E) of PPARα in epidermal keratinocytes and dermal macrophages in different samples (n = 5-7 cases/group). Dashed boxes in panel D are enlarged on right. Brown arrows indicate PPARα expression in subcutaneous macrophages. **(F)** *Ppara* mRNA expression in mouse skin following treatment with PLX3397 (1 mM), L-IMQ (20.8 mg/d), rCSF1/rIL-34 (0.5 μg), or combinations (n = 4-6 mice/group). **(H)** *Ppara* expression in peritoneal macrophages treated with PLX3397 (0.5 μM), IMQ (10 μg/mL), rCSF1/rIL-34 (50 ng/mL) (n = 4-6 wells/group). *P < 0.05, **P < 0.01, ***P < 0.001, ****P < 0.0001 *vs*. HC or CON group; ^#^P < 0.05, ^##^P < 0.01 *vs*. PN or IMQ group; ^$^P < 0.05, ^$$^P < 0.01 *vs*. L-IMQ.

To delineate the downstream effects of CSF1R signaling, we performed KEGG pathway analysis of DEGs in *Csf1r⁺/⁻* versus WT IMQ-treated mice, which identified the PPAR signaling pathway as the most significantly enriched pathway (Figure 8A). Among the 33 PPAR-related DEGs (ko03320) (Figure 8B), *Ppara* and *Pparg* were markedly suppressed in WT IMQ skin but robustly restored in *Csf1r*^+/-^ mice, indicating a CSF1R-dependent repression of PPAR signaling.

This PPAR suppression was conserved in human psoriasis, as bulk RNA-seq analysis of lesional skin (GSE13355) revealed a pronounced downregulation of *PPARα* compared with non-lesional controls (P < 0.0001; Figure 8C). Importantly, sex-stratified scRNA-seq reanalysis demonstrated that *PPARα* expression followed a consistent regulatory direction in both male and female patients, excluding sex bias as a confounding factor (Figure S6A). Moreover, *PPARα* expression was preferentially enriched within the pathogenic CSF1R^high^ myeloid compartment, male patients exhibited significantly lower expression levels of this protective factor compared to females (Figure S6B). This positions PPARα as a downstream node specifically suppressed during the male-biased amplification of pathogenic signaling.

Spatial transcriptomic and immunofluorescence analyses further localized *PPARα* expression to epidermal keratinocytes and dermal macrophages in healthy skin, with signal intensity reduced by approximately 60-75% across distinct psoriasis subtypes (Figure 8D-E). Functional validation revealed that pharmacological CSF1R inhibition with PLX3397 effectively restored IMQ-induced *Ppara* suppression *in vivo* (Figure 8F), whereas exogenous rCSF1 or rIL-34 synergized with low-dose IMQ to further suppress *Ppara* expression. Consistently, *in vitro* stimulation of macrophages with IMQ, rCSF1, or rIL-34 reduced *Ppara* expression, an effect that was fully reversed by CSF1R blockade (Figure 8G). Together, these findings establish CSF1R activation as a direct and conserved repressor of PPARα signaling in both myeloid and epithelial compartments, thereby amplifying inflammatory programs through disruption of this key immunoregulatory checkpoint.

### PPARα activation is essential for the anti-inflammatory effects of CSF1R blockade

**Figure 9:**
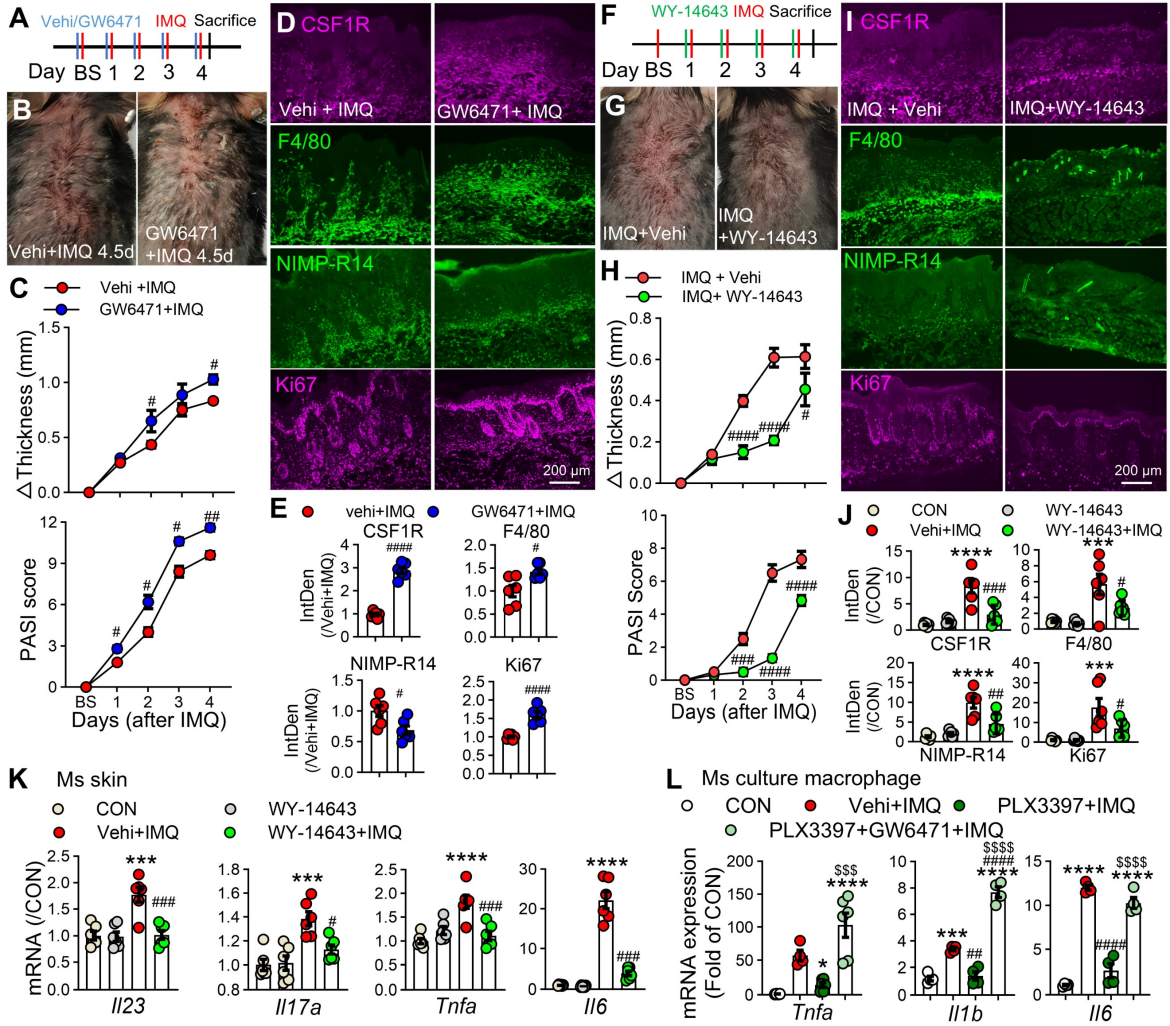
** The CSF1R-PPARα axis orchestrates inflammation. (A)** Schematic diagram of subcutaneous PPARα antagonist GW6471 (10 mM/100 μL) or vehicle administration 30 min prior to IMQ treatment. **(B)** Representative skin phenotypes at IMQ 4.5d after treatment with GW6471 or vehicle. **(C)** Skin thickness and PASI scores in GW6471-treated and vehicle-treated mice compared to the Vehi + IMQ group (n = 5 mice/group). **(D, E)** IF images (D) and quantification (E) of CSF1R^+^F4/80^+^ macrophages and Ki67^+^ proliferating cells (n = 3 mice/group, 2 sections/mouse). **(F)** Schematic of subcutaneous PPARα agonist WY-14643 (10 mM/100 μL) administration in IMQ model. **(G)** Representative skin phenotypes at IMQ 4.5d after treatment with WY-14643 or vehicle.** (H)** Skin thickness/PASI with or without WY-14643 treatment to IMQ groups (n = 5-6 mice/group). **(I, J)** IF images (I) and quantification (J) of CSF1R, F4/80, and Ki67 expression in each group.** (K)** qPCR analysis of pro-inflammatory cytokine mRNAs (*Il23a*, *Il17a*, *Tnfa*, *Il6*) in lesional skin (n = 3 mice/group). **(L)** qRT-PCR showing cytokine expression in peritoneal macrophages pretreated with PLX3397 (0.5 μM) ± GW6471 (5 μM) before IMQ stimulation (n = 4-6 samples/group). P < 0.05, *** P < 0.001, **** P < 0.0001 *vs.* CON; ^#^P < 0.05 *vs.* (Vehi) + IMQ.

To define the functional crosstalk between CSF1R and PPARα, we pharmacologically modulated PPARα activity in IMQ-treated mice. Topical administration of the PPARα antagonist GW6471 (10 mM/100 μL) significantly exacerbated IMQ-induced dermatitis, with more severe lesions, increased erythema, and elevated clinical scores compared to vehicle controls (Figure 9A-C). Immunofluorescence analysis revealed that GW6471 treatment promoted CSF1R expression, enhanced macrophage infiltration, and increased cellular proliferation (Figure 9D-E). Conversely, treatment with the PPARα agonist WY-14643 significantly mitigated psoriasis symptoms, reducing skin inflammation, thickness, clinical scores, and suppressing mRNA expression of key pro-inflammatory cytokines (*Il23*, *Il17a*, *Tnfa*, *Il1b*, *Il6*) in lesional skin (Figure 9F-K). We next investigated whether PPARα activity is functionally integrated downstream of CSF1R signaling. Pretreatment with the CSF1R inhibitor PLX3397 significantly reduced IMQ-induced overexpression of *Tnfa*, *Il1b*, and *Il6* in cultured peritoneal macrophages. Crucially, this anti-inflammatory effect of PLX3397 was completely abolished by PPARα antagonist GW6471 co-treatment (*Tnfa*: ^$$$^P < 0.001, *Il1b* & *Il6*: ^$$$$^P < 0.0001) (Figure 9L). This definitive functional evidence establishes PPARα as a non-redundant downstream effector of CSF1R-directed therapy, defining a unidirectional CSF1R-PPARα axis where CSF1R inhibition relieves PPARα repression to restore anti-inflammatory signaling.

## Discussion

Our study establishes that CSF1R⁺ myeloid cells, and in particular a CSF1R^high^ subset, function as spatially organized pathogenic hubs that drive activation of the IL-23/IL-17 axis across cutaneous and systemic compartments in psoriasis. Using integrated human and murine multi-omics and functional validation, we show that CSF1R is selectively enriched in pro-inflammatory macrophages and specific dendritic cell subsets, which are maintained in a pathological state by dysregulated CSF1 and IL-34 signaling from keratinocytes and stromal cells 42, 43. Importantly, systemic CSF1R inhibition not only depletes these pathogenic reservoirs in skin and blood but also reactivates PPARα-mediated resolution programs, resulting in sustained suppression of inflammation. These findings define the CSF1R-PPARα axis as a central mechanistic link between persistent MPS-driven inflammation and failed resolution in psoriasis.

The pathogenic relevance of myeloid heterogeneity in psoriasis is increasingly recognized 44, 45. Consistent with prior imaging studies in *Csf1r*-reporter mice 46, our spatial and single-cell analyses reveal a marked expansion of CSF1R^high^ MPS cells in lesional skin, with minimal representation in homeostatic compartments. These cells—comprising IL-1β⁺ and IL-23⁺ inflammatory macrophages, cDC2, pDCs, and inflammatory monocytes—show strong spatial associations with psoriatic cytokines and proliferative signatures 47. We further identify circulating CSF1R^high^ monocytes in patients and IMQ mice that amplify systemic inflammation, supporting a model a model in which CSF1R^high^ MPS hubs act as upstream organizers of the IL-23/IL-17 cascade rather than passive responders 48. Although IL-17A is canonically produced by Th17 and Tc17 cells, extensive evidence indicates that myeloid cells establish the inflammatory niches that recruit, license, and sustain IL-17-producing T cells through IL-23, IL-1β, and TNF signaling 7, 8, 48. Accordingly, enrichment of bulk CD4⁺ or CD8⁺ T-cell populations—which includes naïve and regulatory subsets—does not necessarily predict peak tissue IL-17A levels. Instead, the strong association between CSF1R^high^ MPS hubs and IL-17-oriented cytokine landscapes supports a hierarchical model in which myeloid hubs organize pathogenic circuits upstream of effector T cells. This conceptual framework provides a mechanistic rationale for targeting CSF1R^high^ MPS hubs to dismantle IL-17-driven inflammatory architecture at its source, rather than solely neutralizing downstream effector cytokines.

Beyond cellular expansion, we delineate a compartmentalized dysregulation of CSF1R ligands within the psoriatic microenvironment. In healthy skin, CSF1 is primarily produced by fibroblasts and B cells, while IL-34 is abundant in keratinocytes and melanocytes 49. In psoriasis, CSF1 shifts to inflammatory MPS cells, creating autocrine loops that perpetuate activation, while IL-34 is suppressed. This spatiotemporal ligand dysregulation suggests a model wherein CSF1 sustains pro-inflammatory myeloid activation at the core of lesions, while IL-34 may facilitate peripheral lesion propagation via immune-epithelial crosstalk, potentially through receptors like Ptprz1 and Syndecan-1 50 —a hypothesis meriting further study. The biphasic expression of *Csf1*/*Csf1r* post-IMQ reflects waves of monocyte recruitment and local proliferation, reinforcing the notion of self-sustaining pathogenic circuits. Clinically, elevated serum CSF1/IL-34 in PsA patients 51, 52 may fuel a feed-forward loop, enhancing vascular leakage 53 and skin infiltration of CSF1R⁺ monocytes that subsequently suppress PPARα-managed resolution pathways.

The systemic nature of these amplification loops necessitates therapeutic strategies that operate across compartments. We demonstrate that systemic CSF1R inhibition is vastly superior to local intervention, markedly reducing skin thickness, clinical scores, and levels of circulating CSF1R⁺CD45⁺ cells. Local inhibition offers only transient relief, failing to eliminate circulating precursors or deep dermal hubs. In contrast, systemic blockade depletes both tissue-resident and blood-borne CSF1R⁺ MPS cells, effectively disrupting the recruitment-proliferation cycle. This underscores the imperative for early and sustained systemic intervention to dismantle these pathogenic networks, potentially addressing comorbidities unaffected by current biologics 38.

Sexual dimorphism in psoriasis is widely recognized, with male patients often exhibiting higher objective measures of disease severity such as PASI and body surface area involvement despite similar overall prevalence between sexes 54-56. Our protein-level analysis of human skin confirmed that core CSF1R pathway components and downstream inflammatory mediators are upregulated in both male and female psoriasis patients, with a significantly greater magnitude of induction in male skin. scRNA-seq reanalysis revealed that while the overall abundance of MPS cells was comparable, male patients exhibit significantly higher expression levels of *CSF1R* within the pathogenic myeloid subsets. Furthermore, we observed a sex-specific deficit in the resolution machinery: the protective factor *PPARα* was retained at significantly lower levels in the pathogenic MPS hubs of male patients compared to females. These findings suggest that a “double-hit” mechanism—comprising amplified pathogenic CSF1R signaling and defective PPARα-mediated resolution—underlies the observed male bias in clinical severity. Importantly, the identification of this conserved yet variable CSF1R-PPARα pathogenic axis across sexes supports the generalizability of our mechanistic model. This framework reconciles shared core inflammatory mechanisms with sex-linked differences in disease burden and highlights the translational relevance of our human analyses.

A pivotal mechanistic discovery of our study is that CSF1R activation directly suppresses PPARα, a master regulator of inflammatory resolution and metabolic homeostasis 17, 57. PPARα is known to govern epidermal differentiation, lipid metabolism, and immune restraint in the skin, and its downregulation has been repeatedly documented in inflammatory dermatoses, including psoriasis 58, 59. Comprehensive analyses have demonstrated that impaired PPARα signaling compromises keratinocyte differentiation 60 and barrier integrity 61, enhances dendritic cell-driven Th17 priming, and skews macrophages toward a pro-inflammatory metabolic state 62, 63. Despite this consensus, the upstream signals enforcing PPARα suppression in lesional skin have remained undefined. Our data directly address this gap by identifying CSF1R signaling as a previously unrecognized upstream repressor of PPARα in psoriatic lesions. We show that PPARα is markedly downregulated across both myeloid and epithelial compartments, and that genetic or pharmacological inhibition of CSF1R restores PPARα expression and resolution-associated metabolic programs. Importantly, the anti-inflammatory effects of CSF1R blockade were completely abolished by PPARα antagonism, functionally establishing a unidirectional CSF1R-PPARα axis. These findings provide a mechanistic explanation for prior observations of PPARα dysfunction in psoriasis and support a “dual-hit” model in which CSF1R ligands simultaneously drive pathogenic myeloid expansion and metabolically lock inflammatory cells in a non-resolving state through repression of PPARα.

Our findings challenge the prevailing cytokine-centric therapeutic paradigm 64 by positioning CSF1R^high^ MPS hubs as upstream organizers of pathology and identifying PPARα suppression as a core mechanism of failed resolution. This supports a new treatment strategy that concurrently depletes pathogenic myeloid cells and reactivates endogenous resolution programs. This approach directly addresses two major shortcomings of current biologics 65, 66: their inability to fully eliminate myeloid precursors and their failure to restore native anti-inflammatory mechanisms. The superior efficacy of systemic CSF1R targeting 67, 68, combined with the essential role of PPARα, provides a compelling rationale for dual-mechanism therapies co-targeting both pathways to achieve durable disease control 69, particularly in severe and treatment-resistant subtypes.

Our study has several limitations. First, the IMQ-induced murine model recapitulates key IL-23/IL-17-driven inflammatory and epidermal hyperproliferative features but represents an acute inflammatory setting and does not fully model the chronic, relapsing-remitting course of human psoriasis. As such, extrapolation to long-term disease memory and relapse should be interpreted with caution. Second, although the *Cx3cr1^CreER^* driver enabled efficient targeting of CSF1R⁺ pathogenic MPS hubs, its activity spans monocytes, macrophages, and selected dendritic cell subsets. Consequently, our genetic ablation reflects disruption of the broader CSF1R-expressing MPS continuum, rather than a single defined lineage. This design was intentional, as our data support a hub-based, multicellular model of myeloid-driven inflammation; nevertheless, future studies employing more restricted or combinatorial Cre strategies will be valuable to further refine cell-type-specific contributions. Finally, while our preclinical findings provide a mechanistic rationale for systemic CSF1R targeting, translation to patients will require careful evaluation of long-term safety, given the essential homeostatic functions of tissue-resident macrophages. Approaches combining temporal control, partial inhibition, or adjunctive therapies may help balance efficacy and safety in future clinical applications.

In conclusion, building upon foundational work such as Wang et al. (2019) 14, our study firmly establishes pathogenic CSF1R^high^ myeloid cells as critical drivers of psoriatic inflammation. We extend this paradigm by defining the spatial architecture of CSF1R ligand expression, elucidating the CSF1R-PPARα axis as a core pathologic circuit, and demonstrating the necessity of systemic targeting for durable resolution. Our findings advocate for a strategic shift away from mere cytokine blockade 64 and toward therapeutic strategies that simultaneously disrupt pathogenic myeloid circuits and restore endogenous resolution programs.

## Supplementary Material

Supplementary figures and tables.

## Abbreviations

CKO: Conditional knockout
CSF1R: Colony-stimulating factor 1 receptor
DC: Dendritic cell
DEG: Differentially expressed gene
EP: Erythrodermic psoriasis
GPP: Generalized pustular psoriasis
HC: Healthy control
IF: Immunofluorescence
IHC: Immunohistochemistry
IL: Interleukin
IMQ: Imiquimod
LC: Langerhans cell
Mo: Monocyte
MPS: Mononuclear phagocyte system
Mφ: Macrophage
PASI: Psoriasis Area and Severity Index
NPN: Non-lesional skin
PPARα: Peroxisome proliferator-activated receptor alpha
PS: Psoriatic lesional skin
PV: Plaque psoriasis
qRT-PCR: Quantitative reverse transcription polymerase chain reaction
scRNA-seq: Single-cell RNA sequencing
TNF-α: Tumor necrosis factor alpha
WT: Wild-type

## References

[1] Parisi R, Iskandar IYK, Kontopantelis E, Augustin M, Griffiths CEM, Ashcroft DM (2020). National, regional, and worldwide epidemiology of psoriasis: systematic analysis and modelling study. BMJ.

[2] Guo J, Zhang H, Lin W, Lu L, Su J, Chen X Signaling pathways, targeted therapies for psoriasis Signal Transduct Target Ther. 2023; 8: 437.

[3] Griffiths CEM, Armstrong AW, Gudjonsson JE, Barker J Psoriasis Lancet. 2021; 397: 1301-15.

[4] Kokolakis G, Warren RB, Strober B, Blauvelt A, Puig L, Morita A (2023). Bimekizumab efficacy and safety in patients with moderate-to-severe plaque psoriasis who switched from adalimumab, ustekinumab or secukinumab: results from phase III/IIIb trials. Br J Dermatol.

[5] Furue K, Ito T, Furue M Differential efficacy of biologic treatments targeting the TNF-alpha/IL-23/IL-17 axis in psoriasis, psoriatic arthritis Cytokine. 2018; 111: 182-8.

[6] Mehta H, Mashiko S, Angsana J, Rubio M, Hsieh YM, Maari C (2021). Differential Changes in Inflammatory Mononuclear Phagocyte and T-Cell Profiles within Psoriatic Skin during Treatment with Guselkumab vs. Secukinumab. J Invest Dermatol.

[7] Kamata M, Tada Y Dendritic Cells, Macrophages in the Pathogenesis of Psoriasis Front Immunol. 2022; 13: 941071.

[8] Brunner PM, Koszik F, Reininger B, Kalb ML, Bauer W, Stingl G Infliximab induces downregulation of the IL-12/IL-23 axis in 6-sulfo-LacNac (slan)+ dendritic cells, macrophages J Allergy Clin Immunol. 2013; 132: 1184-93 e8.

[9] Hansel A, Gunther C, Ingwersen J, Starke J, Schmitz M, Bachmann M (2011). Human slan (6-sulfo LacNAc) dendritic cells are inflammatory dermal dendritic cells in psoriasis and drive strong TH17/TH1 T-cell responses. J Allergy Clin Immunol.

[10] Grabert K, Sehgal A, Irvine KM, Wollscheid-Lengeling E, Ozdemir DD, Stables J (2020). A Transgenic Line That Reports CSF1R Protein Expression Provides a Definitive Marker for the Mouse Mononuclear Phagocyte System. J Immunol.

[11] Orsenigo F, Stewart A, Hammer CP, Clarke E, Simpkin D, Attia H (2024). Unifying considerations and evidence of macrophage activation mosaicism through human CSF1R and M1/M2 genes. Cell Rep.

[12] Hamilton JA, Achuthan A Colony stimulating factors, myeloid cell biology in health, disease Trends Immunol. 2013; 34: 81-9.

[13] Rojo R, Raper A, Ozdemir DD, Lefevre L, Grabert K, Wollscheid-Lengeling E (2019). Deletion of a Csf1r enhancer selectively impacts CSF1R expression and development of tissue macrophage populations. Nat Commun.

[14] Wang Y, Edelmayer R, Wetter J, Salte K, Gauvin D, Leys L (2019). Monocytes/Macrophages play a pathogenic role in IL-23 mediated psoriasis-like skin inflammation. Sci Rep.

[15] Gharzeddine K, Gonzalez Prieto C, Malier M, Hennot C, Grespan R, Yamaryo-Botte Y (2024). Metabolic reprogramming of hypoxic tumor-associated macrophages through CSF-1R targeting favors treatment efficiency in colorectal cancers. J Immunother Cancer.

[16] Bougarne N, Weyers B, Desmet SJ, Deckers J, Ray DW, Staels B (2018). Molecular Actions of PPARalpha in Lipid Metabolism and Inflammation. Endocr Rev.

[17] Babaev VR, Ishiguro H, Ding L, Yancey PG, Dove DE, Kovacs WJ (2007). Macrophage expression of peroxisome proliferator-activated receptor-alpha reduces atherosclerosis in low-density lipoprotein receptor-deficient mice. Circulation.

[18] Westergaard M, Henningsen J, Johansen C, Rasmussen S, Svendsen ML, Jensen UB (2003). Expression and localization of peroxisome proliferator-activated receptors and nuclear factor kappaB in normal and lesional psoriatic skin. J Invest Dermatol.

[19] Yona S, Kim KW, Wolf Y, Mildner A, Varol D, Breker M (2013). Fate mapping reveals origins and dynamics of monocytes and tissue macrophages under homeostasis. Immunity.

[20] Molawi K, Wolf Y, Kandalla PK, Favret J, Hagemeyer N, Frenzel K (2014). Progressive replacement of embryo-derived cardiac macrophages with age. J Exp Med.

[21] Epelman S, Lavine KJ, Randolph GJ Origin, functions of tissue macrophages Immunity. 2014; 41: 21-35.

[22] Guilliams M, Ginhoux F, Jakubzick C, Naik SH, Onai N, Schraml BU (2014). Dendritic cells, monocytes and macrophages: a unified nomenclature based on ontogeny. Nat Rev Immunol.

[23] van der Fits L, Mourits S, Voerman JS, Kant M, Boon L, Laman JD (2009). Imiquimod-induced psoriasis-like skin inflammation in mice is mediated via the IL-23/IL-17 axis. J Immunol.

[24] Chen Y, Yan Y, Liu H, Qiu F, Liang CL, Zhang Q (2020). Dihydroartemisinin ameliorates psoriatic skin inflammation and its relapse by diminishing CD8(+) T-cell memory in wild-type and humanized mice. Theranostics.

[25] Kim D, Langmead B, Salzberg SL HISAT (2015). a fast spliced aligner with low memory requirements. Nat Methods.

[26] Pertea M, Pertea GM, Antonescu CM, Chang TC, Mendell JT, Salzberg SL StringTie enables improved reconstruction of a transcriptome from RNA-seq reads Nat Biotechnol. 2015; 33: 290-5.

[27] Wang L, Feng Z, Wang X, Wang X, Zhang X DEGseq (2010). an R package for identifying differentially expressed genes from RNA-seq data. Bioinformatics.

[28] Chu T, Wang Z, Pe'er D, Danko CG Cell type, gene expression deconvolution with BayesPrism enables Bayesian integrative analysis across bulk, single-cell RNA sequencing in oncology Nat Cancer. 2022; 3: 505-17.

[29] Singh TP, Zhang HH, Borek I, Wolf P, Hedrick MN, Singh SP (2016). Monocyte-derived inflammatory Langerhans cells and dermal dendritic cells mediate psoriasis-like inflammation. Nat Commun.

[30] Sakamoto K, Goel S, Funakoshi A, Honda T, Nagao K Flow cytometry analysis of the subpopulations of mouse keratinocytes, skin immune cells STAR Protoc. 2022; 3: 101052.

[31] Hou Y, Zhu L, Tian H, Sun HX, Wang R, Zhang L (2018). IL-23-induced macrophage polarization and its pathological roles in mice with imiquimod-induced psoriasis. Protein Cell.

[32] Tang-Huau TL, Gueguen P, Goudot C, Durand M, Bohec M, Baulande S (2018). Human *in vivo*-generated monocyte-derived dendritic cells and macrophages cross-present antigens through a vacuolar pathway. Nat Commun.

[33] Schulz C, Gomez Perdiguero E, Chorro L, Szabo-Rogers H, Cagnard N, Kierdorf K (2012). A lineage of myeloid cells independent of Myb and hematopoietic stem cells. Science.

[34] Gautier EL, Shay T, Miller J, Greter M, Jakubzick C, Ivanov S (2012). Gene-expression profiles and transcriptional regulatory pathways that underlie the identity and diversity of mouse tissue macrophages. Nat Immunol.

[35] Zhu L, Yang T, Li L, Sun L, Hou Y, Hu X (2014). TSC1 controls macrophage polarization to prevent inflammatory disease. Nat Commun.

[36] Combes TW, Orsenigo F, Stewart A, Mendis A, Dunn-Walters D, Gordon S (2021). CSF1R defines the mononuclear phagocyte system lineage in human blood in health and COVID-19. Immunother Adv.

[37] Kim J, Lee J, Li X, Lee HS, Kim K, Chaparala V (2023). Single-cell transcriptomics suggest distinct upstream drivers of IL-17A/F in hidradenitis versus psoriasis. J Allergy Clin Immunol.

[38] Korman NJ Management of psoriasis as a systemic disease (2020). what is the evidence?. Br J Dermatol.

[39] Costa MC, Rocha BO, Paixao CS, Oliveira M, Mota L, Carvalho LP Monocyte subpopulations study in patients with plaque psoriasis Med Hypotheses. 2017; 104: 101-3.

[40] Solberg SM, Aarebrot AK, Sarkar I, Petrovic A, Sandvik LF, Bergum B (2021). Mass cytometry analysis of blood immune cells from psoriasis patients on biological therapy. Eur J Immunol.

[41] Qin P, Ho FK, Celis-Morales CA, Pell JP Association between systemic inflammation biomarkers and incident cardiovascular disease in 423,701 individuals (2025). evidence from the UK biobank cohort. Cardiovasc Diabetol.

[42] Greter M, Lelios I, Pelczar P, Hoeffel G, Price J, Leboeuf M (2012). Stroma-derived interleukin-34 controls the development and maintenance of langerhans cells and the maintenance of microglia. Immunity.

[43] Wang Y, Szretter KJ, Vermi W, Gilfillan S, Rossini C, Cella M (2012). IL-34 is a tissue-restricted ligand of CSF1R required for the development of Langerhans cells and microglia. Nat Immunol.

[44] Park S, Jang J, Kim HJ, Jung Y Unveiling multifaceted roles of myeloid innate immune cells in the pathogenesis of psoriasis Mol Aspects Med. 2024; 99: 101306.

[45] Reynolds G, Vegh P, Fletcher J, Poyner EFM, Stephenson E, Goh I (2021). Developmental cell programs are co-opted in inflammatory skin disease. Science.

[46] Irvine KM, Caruso M, Cestari MF, Davis GM, Keshvari S, Sehgal A (2020). Analysis of the impact of CSF-1 administration in adult rats using a novel Csf1r-mApple reporter gene. J Leukoc Biol.

[47] Strychalski ML, Brown HS, Bishop SC Cytokine Modulators in Plaque Psoriasis - A Review of Current, Prospective Biologic Therapeutic Approaches JAAD Int. 2022; 9: 82-91.

[48] Pantelyushin S, Haak S, Ingold B, Kulig P, Heppner FL, Navarini AA (2012). Rorgammat+ innate lymphocytes and gammadelta T cells initiate psoriasiform plaque formation in mice. J Clin Invest.

[49] Croquette M, Faugeroux A, Fonlupt C, Godet J, Frouin E, Garcia M (2023). IL-34 Exerts Anti-Inflammatory Activities on Keratinocytes and Is Downregulated in Psoriatic-Inflamed Skin. J Invest Dermatol.

[50] Baghdadi M, Umeyama Y, Hama N, Kobayashi T, Han N, Wada H (2018). Interleukin-34, a comprehensive review. J Leukoc Biol.

[51] Jadon DR, Nightingale AL, McHugh NJ, Lindsay MA, Korendowych E, Sengupta R Serum soluble bone turnover biomarkers in psoriatic arthritis, psoriatic spondyloarthropathy J Rheumatol. 2015; 42: 21-30.

[52] Li J, Liu L, Rui W, Li X, Xuan D, Zheng S (2017). New Interleukins in Psoriasis and Psoriatic Arthritis Patients: The Possible Roles of Interleukin-33 to Interleukin-38 in Disease Activities and Bone Erosions. Dermatology.

[53] Li Q, Pang B, Dang E, Wang G Endothelial Dysfunction in Psoriasis (2024). An Integrative Review. J Invest Dermatol.

[54] Guillet C, Seeli C, Nina M, Maul LV, Maul JT The impact of gender, sex in psoriasis (2022). What to be aware of when treating women with psoriasis. Int J Womens Dermatol.

[55] Wang X, Ma R, Shi R, Qin H, Chen W, Yu Z (2023). Sex differences in the association between plasma polyunsaturated fatty acids levels and moderate-to-severe plaque psoriasis severity: a cross-sectional and longitudinal study. J Transl Med.

[56] Goldburg S, Chen R, Langholff W, Lafferty KP, Gooderham M, de Jong EM (2022). Sex Differences in Moderate to Severe Psoriasis: Analysis of the Psoriasis Longitudinal Assessment and Registry. J Psoriasis Psoriatic Arthritis.

[57] Zandbergen F, Plutzky J PPARalpha in atherosclerosis, inflammation Biochim Biophys Acta. 2007; 1771: 972-82.

[58] Sertznig P, Seifert M, Tilgen W, Reichrath J Peroxisome proliferator-activated receptors (PPARs), the human skin (2008). importance of PPARs in skin physiology and dermatologic diseases. Am J Clin Dermatol.

[59] Lima Ede A, Lima MM, Marques CD, Duarte AL, Pita Ida R, Pita MG Peroxisome proliferator-activated receptor agonists (PPARs) (2013). a promising prospect in the treatment of psoriasis and psoriatic arthritis. An Bras Dermatol.

[60] Dubrac S, Schmuth M PPAR-alpha in cutaneous inflammation Dermatoendocrinol. 2011; 3: 23-6.

[61] Chon SH, Tannahill R, Yao X, Southall MD, Pappas A Keratinocyte differentiation, upregulation of ceramide synthesis induced by an oat lipid extract via the activation of PPAR pathways Exp Dermatol. 2015; 24: 290-5.

[62] Li Y, Li Y, Xu S, Chen Y, Zhou P, Hu T (2022). N-Acylethanolamine acid amidase (NAAA) exacerbates psoriasis inflammation by enhancing dendritic cell (DCs) maturation. Pharmacol Res.

[63] Li Y, Yuan H, Cheng YL, Li QX, Cao R, Li JL (2025). Hydrogen-bonded organic framework for regulating pathogenic autoantigen and oxidative stress in psoriasis treatment. Natl Sci Rev.

[64] Ghoreschi K, Balato A, Enerback C, Sabat R Therapeutics targeting the IL-23, IL-17 pathway in psoriasis Lancet. 2021; 397: 754-66.

[65] Blauvelt A, Kimball AB, Augustin M, Okubo Y, Witte MM, Rodriguez Capriles C (2022). Efficacy and Safety of Mirikizumab in Psoriasis: Results from a 52-Week, Double-Blinded, Placebo-Controlled, Randomised Withdrawal, Phase III Trial (OASIS-1). Br J Dermatol.

[66] Reich K, Armstrong AW, Langley RG, Flavin S, Randazzo B, Li S (2019). Guselkumab versus secukinumab for the treatment of moderate-to-severe psoriasis (ECLIPSE): results from a phase 3, randomised controlled trial. Lancet.

[67] MacDonald KP, Palmer JS, Cronau S, Seppanen E, Olver S, Raffelt NC (2010). An antibody against the colony-stimulating factor 1 receptor depletes the resident subset of monocytes and tissue- and tumor-associated macrophages but does not inhibit inflammation. Blood.

[68] Pyonteck SM, Akkari L, Schuhmacher AJ, Bowman RL, Sevenich L, Quail DF (2013). CSF-1R inhibition alters macrophage polarization and blocks glioma progression. Nat Med.

[69] Liu Z, Guo C, Das SK, Yu X, Pradhan AK, Li X (2021). Engineering T Cells to Express Tumoricidal MDA-7/IL24 Enhances Cancer Immunotherapy. Cancer Res.

