# Supplementary material for: Systemic CSF1R Targeting Depletes Pathogenic MPS Bubs and Ameliorates Psoriasis via PPARα-mediated Resolution
Source: Theranostics. 2026 Jan 30;16(8):4336–57. doi: 10.7150/thno.128248 (PMC12905821; doi:10.7150/thno.128248)
Supplement: Supplementary file 1 — Supplementary figures and tables. [file thnov16p4336s1.pdf]

Systemic CSF1R Ablation Eradicates Pathogenic MPS Hubs and Halts Psoriasis via PPARα Reactivation

Zhen-Jia Lin, Ying Li, Yangyinhui Yu, Mei-Jia Fang, Ying Xiong, Rui Xu, Zhuo Wang, Jun Zhang, Ya-Nan Xu, Jun-Ya Wan, Xiang Ji, Yu-Fan Zheng, Kai-Lang Zhang, Ming Wei, Jun-Tao Zou, Li-Xuan Jia, Hui Zhang[5]*, Chang-Lin Li*, Li-Jun Zhou*, Zhi Tan

Supplementary information includes:

Supplementary Figures:

Figure S1. Reanalysis of single-cell RNA-sequencing (scRNA-seq) of human psoriasis skin samples.
Figure S2. scRNA-seq reanalysis identifies molecular and cellular features in psoriatic mouse skin.
Figure S3. Systemic inflammation in psoriasis patients and IMQ-treated mice.
Figure S4. Sex-associated enrichment of pathogenic CSF1R$^+$ myeloid subsets in human psoriasis.
Figure S5. Local CSF1R inhibition partially alleviates IMQ-induced psoriasiform dermatitis.
Figure S6. Sex-independent enrichment of PPARα expression in pathogenic CSF1Rhigh myeloid populations

Supplementary Tables:

Table S1. Characteristics of healthy control and psoriatic participants
Table S2. Demographic and clinical characteristics of human skin and blood samples
(For details, please refer to the Excel table)
Table S3. Flow cytometry antibodies
Table S4. Rationale for selected subcutaneous doses
Table S5. Primer for qRT-PCR in mouse tissues

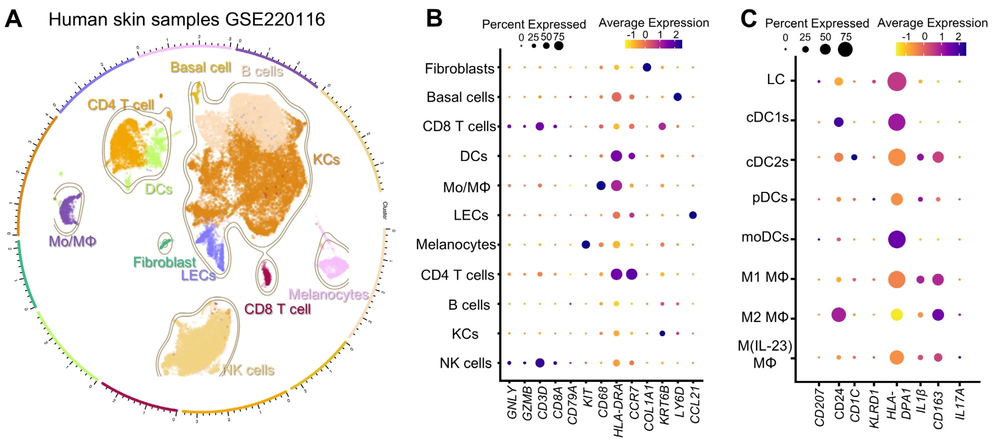

Figure S1. Reanalysis of single-cell RNA-sequencing (scRNA-seq) of human psoriasis skin samples. (A) UMAP plot showing the distribution of 11 distinct cell types in human skin samples from healthy controls (HC; female n = 2, male n = 8) and psoriasis patients (PS, female n = 4, male n = 6) in the GSE220116 dataset. DCs: dendritic cells; KCs: keratinocytes; LECs: lymphatic endothelial cells; Mo/MΦ: monocytes/macrophages. **(B)** Dot plot showing canonical marker genes used to cell-type annotation in panel (A). Dot size indicates the percentage of cells expressing each gene, color intensity reflects average expression. **(C)** Comparison of MPS versus non-MPS cellular proportions between female and male samples. P value was determined by chi-square test. **(D)** Dot plot of marker genes defining 8 distinct MPS subsets, including Mo/MΦ, Langerhans cells (LCs), conventional dendritic cells 1/2 (cDC1s/cDC2s), plasmacytoid dendritic cells (pDCs), monocyte-derived dendritic cells (moDCs).

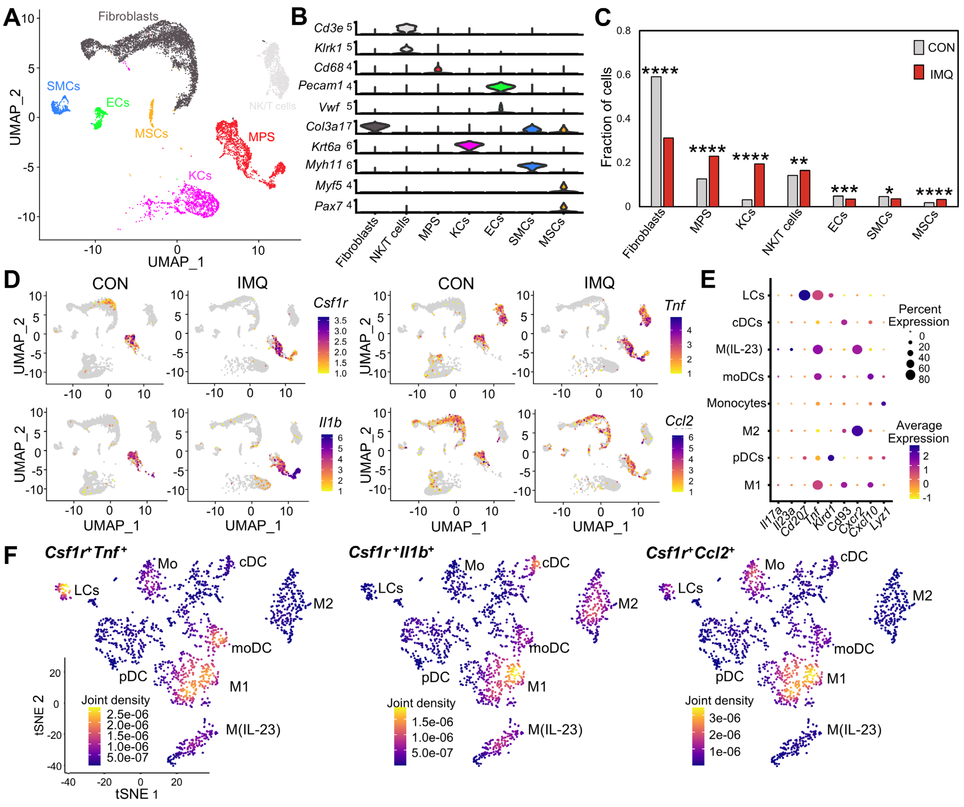

Figure S2. scRNA-seq reanalysis identifies cellular and molecular features in murine psoriatic skin. (A) UMAP embedding of pooled scNRA-seq (GSE230513 and GSE231728; n = 2 controls, n = 2 IMQ) colored by major cell type annotations: including fibroblasts, NK/T cells, MPS, KCs, endothelial cells (ECs), smooth muscle cells (SMCs), and muscular stem cells (MSCs). **(B)** Violin plots of canonical marker genes used for cell type annotation depicted in (A). **(C)** Comparison of cell-type fractions between control (CON) and IMQ groups. *P < 0.05, **P < 0.01, ***P < 0.001, ****P < 0.0001 *vs.* CON. **(D)** UMAP plots visualizing expression of *Csf1r*, *Tnf*, *Il1b*, and *Ccl2* in CON and IMQ groups. **(E)** Dot plots showing subset-defining markers across annotated MPS clusters (dot size = percent of cells expressing the gene; color = average expression). **(F)** tSNE joint-density plots displaying co-expression density of *Csf1r* with *Tnf*, *Il1b*, and *Ccl2* cross MPS subsets; warmer colors indicate higher joint expression density.

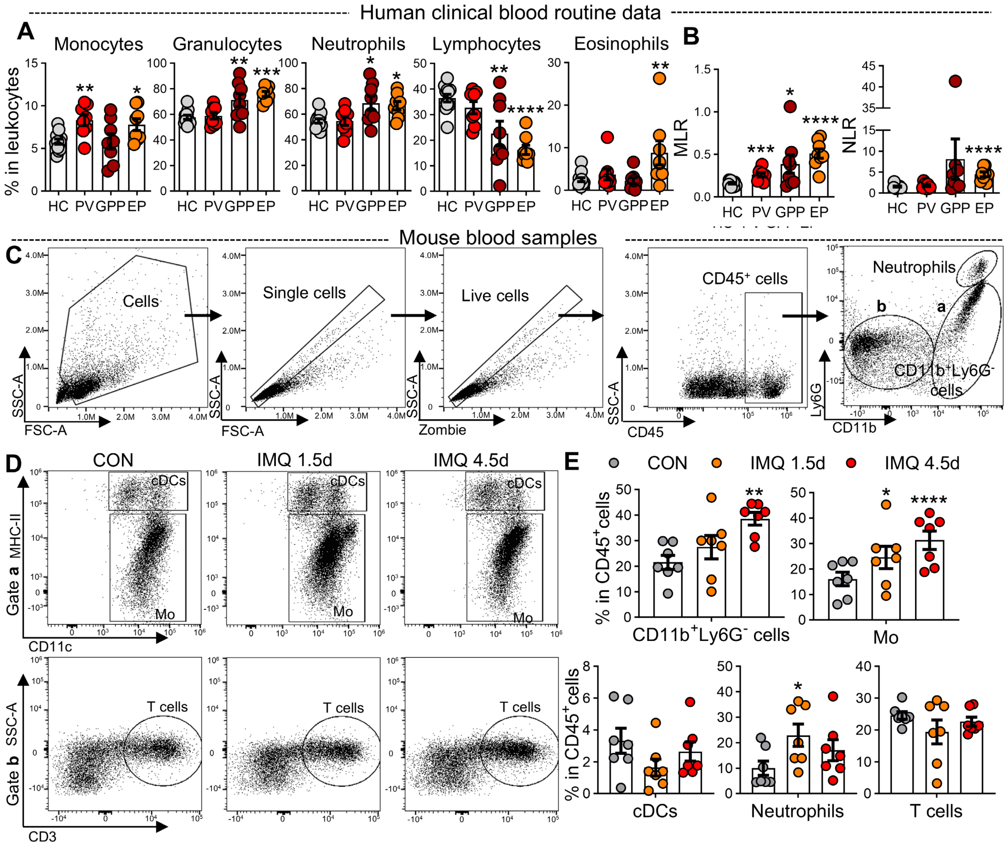

Figure S3. Systemic inflammation in psoriasis patients and IMQ-treated mice. (A, B) The proportion of various types of leukocytes, the monocyte-to-lymphocyte ratio (MLR) and neutrophil-to-lymphocyte ratio (NLR) in peripheral blood of patients with different subtypes of psoriasis. Peripheral blood analysis from a retrospective psoriasis cohort (HC: n = 12, PV/GPP/EP: n = 8/group). Blood was collected during routine clinical visits. Skin biopsies for IHC were provided by a subset of these patients (Fig. 1G, H). **(C)** Flow cytometry gating strategy for immune cell analysis in mouse blood samples from control (CON), IMQ 1.5d, and IMQ 4.5d groups. **(D, E)** Representative flow cytometry dot plots (D) and statistical quantification (E) of $CD11b^{+}Ly6G^{-}$ cells, Mo, cDCs, neutrophils, and T cells in mouse blood at different time points. *P < 0.05, **P < 0.01, ***P < 0.001 *vs.* HC or CON.

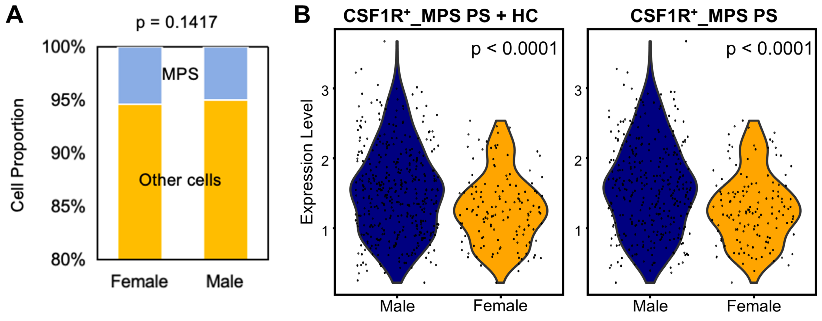

Figure S4. Sex-associated differences in CSF1R expression within pathogenic MPS compartments in human psoriasis. (A) Comparison of mononuclear phagocyte system (MPS) versus non-MPS cellular proportions between female and male samples across healthy control (HC) and psoriasis (PS) conditions in the GSE220116 scRNA-seq dataset. No significant difference in overall MPS abundance was detected between sexes (chi-square test, P = 0.1417). **(B)** Sex-stratified analysis of CSF1R expression within MPS cells. Violin plots show significantly higher CSF1R expression levels in male-derived MPS cells compared to female-derived cells in both the combined HC + PS cohort (left) and PS samples alone (right) (Student's t-test, both P < 0.0001).

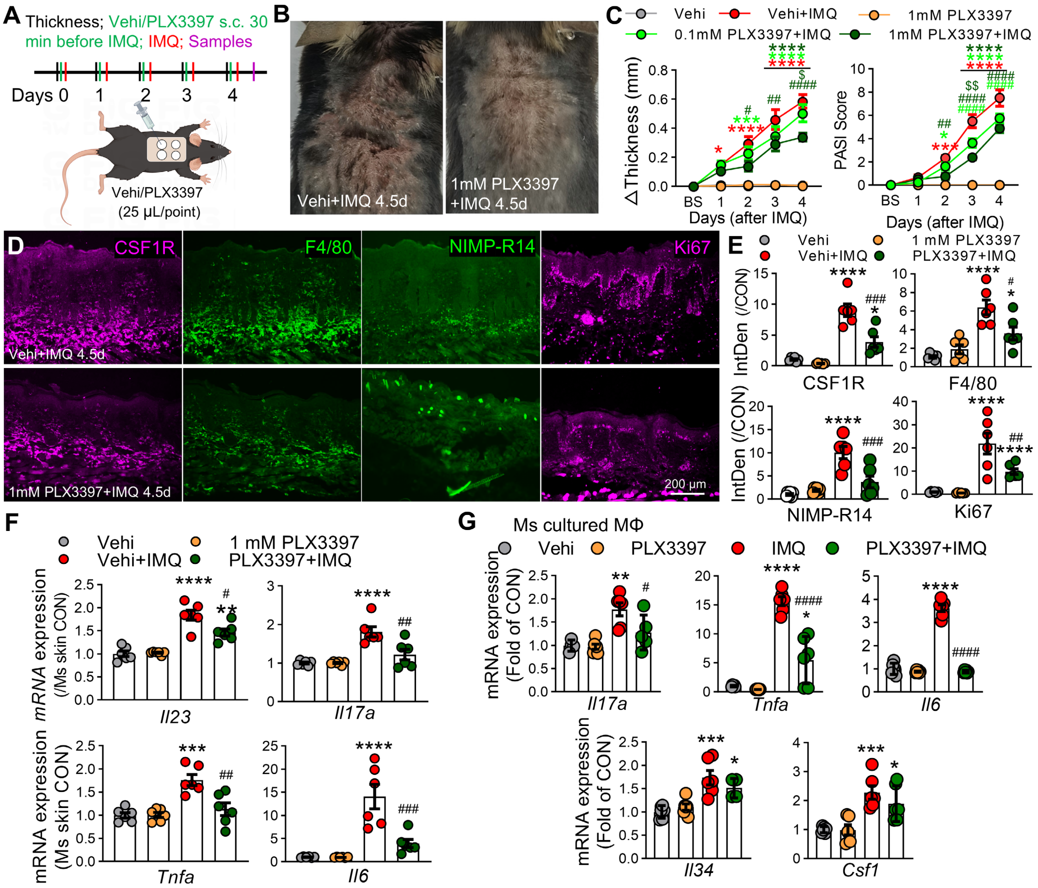

Figure S5. Local CSF1R inhibition partially alleviates IMQ-induced psoriasiform dermatitis. (A) Experimental timeline: Mice received subcutaneous injections of PLX3397 (CSF1R inhibitor; 0.1 or 1 mM, 100 μL total, 4 injection points) or vehicle (Vehi) 30 min prior to daily topical IMQ (62.5 mg/d) or Vaseline (Vehi) for 4.5 days. **(B)** Representative dorsal skin images at IMQ 4.5d. **(C)** Changes in skin fold thickness (left) and PASI scores (right) (n = 6-9 mice/group). **(D, E)** Representative IF images (d) and quantitative analysis (e) of fluorescence signals in skin samples at IMQ 4.5d (n = 3 mice/group, 3 slices/mouse). **(F)** qPCR analysis of inflammatory cytokines mRNA (*Il23*, *Il17a*, *Tnfa*, *Il6*) in skin at IMQ 2.5d (n = 3 mice/group, 2-3 skin samples/mouse). **(G)** Effects of PLX3397 pretreatment on expression of pro-inflammatory cytokine and CSF1R ligand (*Csf1*, *Il34*) mRNA in peritoneal macrophages pretreated with PLX3397 (0.5 μM) or Vehi for 1 h, then stimulated with IMQ (10 μg/mL) for 24 h (n = 4–6 biological replicates/group). *P < 0.05, **P < 0.01, ***P < 0.001, ****P < 0.0001 compared to Vehi; $^{\#}$P < 0.05 *vs.* Vehi + IMQ group; $^{\$}$P < 0.05 *vs.* 0.1 mM PLX3397 + IMQ group.

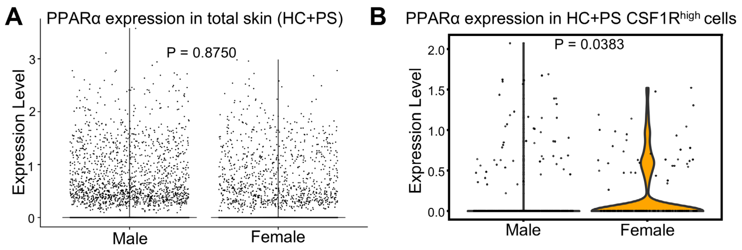

Figure S6. Sex-stratified analysis of PPARα expression in pathogenic CSF1R^{high} MPS cells. (**A**) Comparison of global PPARα expression levels in total skin cells from combined HC and PS samples (GSE220116). No significant global difference was observed between sexes ($P = 0.8750$). (**B**) Violin plot analysis of PPARα expression specifically within the pathogenic CSF1R^{high} MPS subset (incorporating both HC and PS samples). Notably, males exhibited significantly lower expression of PPARα compared to females ($P = 0.0383$).

Table S1. Characteristics of healthy control and psoriatic participants

Groups	Control (n = 16)	Psoriasis (n = 32)
**Gender (male/female)**	10/6	22/10
**Age (years)**	52.44 ± 2.49	43.16 ± 2.44
**Clinical subtypes (PV/GPP/EP)**	N/A	n = 16/8/8

Note: N/A: not applicable. Data are expressed as the number of patients, mean and SEM.

Table S2. Demographic and clinical characteristics of human skin and blood samples (For details, please refer to the Excel table)

Table S3. Flow cytometry antibodies

Target	Clone	Fluorophore	Vendor	Cat. No.	Dilution
**F4/80**	BM8	BUV563	Thermo	365-4801-82	1:100
**Ki67**	B56	BUV805	Thermo	368-5698-82	1:200
**CSF1R**	AFS98	BV421	BioLegend	135513	1:200
**Zombie Viability**	-	BV510	BioLegend	423101	1:400
**EpCAM**	G8.8	BV605	BioLegend	118227	1:100
**TNF-α**	MP6-XT22	BV650	BioLegend	506333	1:100
**Ly6G**	1A8	BV711	BioLegend	127643	1:200
**CD3**	17A2	BV785	BioLegend	100232	1:100
**Ly6C**	HK1.4	AF488	Thermo	53-5932-82	1:200
**IL-1β**	NJTEN3	PerCP-eF710	Thermo	46-7114-82	1:200
**CCL2**	2H5	PE	Thermo	12-7096-82	1:200
**IL-17A**	TC11-18H10	PE-Dazzle594	BioLegend	506938	1:200
**CD11b**	M1/70	PE-Cy5	BioLegend	101210	1:500
**CD64**	X54-5/7.1	PE-Cy7	BioLegend	139314	1:200
**CD11c**	N418	APC	BioLegend	117310	1:200
**MHC-II**	M5/114.15.2	Alexa700	BioLegend	107622	1:200
**CD45**	30-F11	APC-Cy7	BioLegend	103116	1:200
**α-CD16/32 (Fc block)**	2.4G2	-	BioLegend	101320	1:500

Table S4. Rationale for selected subcutaneous doses

Compounds	Representative literature (in-vivo dosing / route)	Representative literature (in-vitro activity)	Our pilot in vivo data (summary)	Dose used in this study (s.c.)
**Recombinant CSF1 (rCSF1)**	CSF1 administration in mice expands monocyte/macrophage populations in vivo (sub-microgram range)(Boissonneault et al., 2009; Sehgal et al., 2021)	Effective at 10–50 ng/mL for macrophage differentiation/activation	0.5 μg/mouse subcutaneously injected (s.c.) enhanced IMQ-induced inflammation consistent with published effects (Figures 5, 8F- G)	0.5 μg/mouse, 100 μL s.c.
**Recombinant IL-34 (rIL-34)**	Effective in vivo range 0.1–1 μg per injection (Munoz-Garcia et al., 2025; Xie et al., 2023)	Typically, active at 25–50 ng/mL in vitro (Munoz-Garcia et al., 2025)	0.5 μg/mouse s.c. augmented pathological changes in the IMQ model, consistent with known biology (Figures 5, 8F- G)	0.5 μg/mouse, 100 μL s.c.
**PLX3397** (CSF1R inhibitor)	Widely used to deplete macrophages in mice at systemic exposures equivalent to ~40–50 mg/kg/day (oral/gavage/chow) (Fujiwara et al., 2021; Merry et al., 2020)	CSF1R inhibition in primary macrophages at sub-μM to low-μM range (Fujiwara et al., 2021)	Dose-response screening (0.1, 1 mM) results showed dose-dependent suppression of psoriasis phenotype, CSF1R signaling and F4/80 expression at 0.1–1 mM with no obvious skin damage (Figure 4)	0.1 or 1 mM, 100 μL s.c. (30 min before IMQ)
**WY-14643** (PPARα agonist)	Commonly used at 10–50 mg/kg in mouse inflammatory /metabolic studies (Yoo et al., 2013)	Potent PPARα activation at ~10–300 μM in cell studies (Neuhaus et al., 2017)	s.c. dosing increased PPARα target gene expression and reduced inflammatory markers with good tolerability (Figure 9)	10 mM, 100 μL s.c. (Day2–5)
**GW6471** (PPARα antagonist)	Frequently used at ~10–20 mg/kg in mice (IP or local routes) (Abu Aboud et al., 2015)	Inhibits PPARα signaling at 1–20 μM (Orozco Morales et al., 2022)	s.c. dosing produced expected inhibition of PPARα targets and reversed WY-14643 effects without toxicity (Figure 9)	10 mM, 100 μL s.c. (Day1–5)

Note: For small-molecule compounds administered locally by subcutaneous injection, doses are expressed as molar concentration to reflect local tissue exposure rather than systemic mg/kg equivalents. Injection volume was fixed at 100 μL for all s.c. administrations.

Table S5. Primer for qRT-PCR in mouse tissues

Primers	Forwards (5'-3')	Reverse (3'-5')
Tnfa	CATCTTCTCAAAATTCGAGTGAC	TGGGAGTAGACAAGGTACAACCC
Il6	GAGGATACCACTCCCAACAGACC	AAGTGCATCATCGTTGTTCATACA
Csf1r	TGTCATCGAGCCTAGTGGC	CGGGAGATTCAGGGTCCAAG
Il23	TTCAGATGGGCATGAATGTTTCT	CCAAATCCGAGCTGTTGTTCTAT
Il17a	TCAGCGTGTCCAAACACTGAG	CGCCAAGGGAGTTAAAGACTT
Ppara	AGAGCCCCATCTGTCCTCTC	ACTGGTAGTCTGCAAAACCAAA
Ppard	TCCATCGTCAACAAAGACGGG	ACTTGGGCTCAATGATGTCAC
Il1b	TGGAAAAGCGGTTTGTCTTC	TACCAGTTGGGGAACTCTGC
Csf1	FTCCCTGAGTCTGTCTTCCAC	CGATGGCTCCTCCACTTC
Il34	TTGCTGTAAACAAAGCCCCAT	CCGAGACAAAGGGTACACATTT
Gapdh	GGCCTTCCGTGTTCCTAC	TGTCATCATATCTGGCAGGTT
